# Evaluating the Antibiotic Susceptibility of *Chlamydia* – New Approaches for *in Vitro* Assays

**DOI:** 10.3389/fmicb.2018.01414

**Published:** 2018-07-03

**Authors:** Hanna Marti, Nicole Borel, Deborah Dean, Cory A. Leonard

**Affiliations:** ^1^Institute of Veterinary Pathology, Vetsuisse Faculty, University of Zurich, Zurich, Switzerland; ^2^Center for Immunobiology and Vaccine Development, UCSF Benioff Children’s Hospital Oakland Research Institute, Oakland, CA, United States; ^3^Joint Graduate Program in Bioengineering, University of California, San Francisco, San Francisco, CA, United States; ^4^Joint Graduate Program in Bioengineering, University of California, Berkeley, Berkeley, CA, United States; ^5^School of Medicine, University of California, San Francisco, San Francisco, CA, United States

**Keywords:** *Chlamydia*, antibiotic resistance, new diagnostic approaches, antibiotic susceptibility assay, resistance screen, minimal inhibitory concentration, recovery assay

## Abstract

Pigs are the natural hosts of *Chlamydia suis*, the only *Chlamydia* species known to spontaneously acquire homotypic resistance conferred by a class C tetracycline resistance gene. Various susceptibility assays have existed for several years, but there is no widely accepted, standardized assay to determine chlamydial antibiotic susceptibility. In this study, we developed new approaches to determine the *in vitro* susceptibility of *Chlamydia* to different antibiotics in view of existing protocols. Specifically, the minimal inhibitory concentration (MIC) is based on a consensus of both inclusion number reduction and alteration of inclusion size and morphology upon antibiotic exposure. In addition to these, we employed a recovery assay, allowing observation of the chlamydial response to drug removal and subsequent recovery, as compared to both continued exposure and to the unexposed control. We propose a simple and fast screening method to detect tetracycline resistant *C. suis* strains within 2 to 3 days with minimal use of consumables. For proof of principle, we evaluated the susceptibility of three *C. suis* field strains and the reference strain S45/6 to tetracycline, sulfamethoxazole, and penicillin, antibiotics commonly used to prevent respiratory and gastrointestinal diseases on fattening pig farms. We found that tetracycline sensitive strains can easily be distinguished from resistant strains using the evaluation parameters proposed in this study. Moreover, we report that S45/6 is sensitive to sulfamethoxazole while all evaluated *C. suis* field strains showed some degree of sulfamethoxazole resistance. Finally, we confirm that Penicillin G induces the chlamydial stress response in all evaluated *C. suis* strains.

## Introduction

*In vitro* susceptibility assays are essential to detect antibiotic resistance in *Chlamydia*. Protocols have existed since the 1980s (Henning and Krauss, 1986), but unlike the standardized tests that are in place for extracellular and facultative intracellular bacteria (CLSI, 2012; Jorgensen and Turnidge, 2015), there is no clear consensus among the research community for antibiotic resistance determination in obligate intracellular bacteria such as *Chlamydia*. **Table 1** summarizes previously reported protocols and/or definitions to determine the antibiotic susceptibility in *Chlamydia*.

**Table 1:** List of reported antibiotic susceptibility assays.

Group/reference	Definition of MIC and MBC/MCC/MLC	Cells	Species
Suchland et al., 2003	**MIC**: “The transition point MIC (MIC_TP_) was defined as the concentration of drug in which **90% or more of the inclusions were altered in size and morphology**. The MIC was defined as the concentration of drug that is one twofold dilution more concentrated than the MIC_TP_.” (p. 637) **MCC**: “The lowest concentration of drug that **produced no morphologically normal inclusions by one freeze-thaw passage** (…) in antimicrobial-free medium.” (p. 637)	HeLa, BGMK, HEp-2, Vero	CT^1^, Cpsi^2^, Cpne^3^

Donati et al., 2010	**MIC**: “The lowest concentration that **reduced the number of inclusions more than 90%** compared with the level for drug-free controls.” (p. 5380) **MBC**: “The MBC concentration was measured by aspirating the antibiotic-containing medium, washing the monolayer twice with PBS, and incubating it in (…) antibiotic-free medium for 48 h at 35°C. (…). The MBC was the lowest concentration of the drug **reducing more than 90% demonstrable inclusions after monolayers were re-incubated in antimicrobial-free medium**.” (p. 5380)	LLC-MK2	CT^1^

Japan Society of Chemotherapy, 1992a,b	**MIC**: “The lowest concentration, which **completely inhibits inclusion body formation**.” (p. 309) **MLC**: “The lowest concentration, which **completely inhibits the reformation of chlamydial inclusion** in infected HeLa 229 cells even **after elimination of the drug** from the culture medium (after 24 h).” (p. 318)	HeLa, McCoy	CT^1^, Cpsi^2^, Cpne^3^

Hammerschlag, 1982; Ehret and Judson, 1988	**MIC**: “The MIC should be defined as **no inclusions seen**.” (Ehret, p. 1298; Hammerschlag, p. 500) **MBC**: “(…) the MBC should be defined as **no inclusion seen on passaging**.” (Ehret, p. 1298)	McCoy	CT^1^

Webberley et al., 1987	**MIC**: “The MIC was defined as the lowest concentration of antibiotic that **inhibited inclusion development** after 48 h.” (p. 409) **MLC**: “(…) the lowest concentration that **prevented the reappearance of inclusions after re-incubation** in antibiotic free media.” (p. 409)	McCoy	Cpne^3^

Agacfidan et al., 1993	**MIC**: “The MIC for *Chlamydia* was defined as the concentration of antibiotic that allowed **no inclusions** on the first passage.” (p. 1993) **MCC**: “The MCC was defined as the lowest concentration that allowed **no inclusions in a further passage** in the absence of antibiotics.” (p. 1993)	McCoy	CT^1^

Henning and Krauss, 1986	**MIC (MHK, Minimale Hemmkonzentration)**: “The lowest concentration where there are **no inclusions**.” (p. 448) **MBC (Washed and reincubated, W & R)**: “The lowest concentration of drug that produced **no inclusions after removal of the drug** and several washing steps during incubation in drug-free medium.” (p. 448)	BGM, McCoy	Cpsi^2^

^*1*^*C. trachomatis*, ^*2*^*C. psittaci*, ^*3*^*C. pneumoniae.*

The aim of this study was to investigate new approaches to determine the *in vitro* susceptibility of *Chlamydia* to different antibiotics. To achieve this goal, the parameters were defined based on consideration of existing protocols (**Table 1**). *Chlamydia suis* was selected as a model for other *Chlamydia* species because of its high degree of genetic diversity and because it is the only example of homotypic resistance to antibiotics among the *Chlamydia*, which is conferred by an acquired resistance gene (Lenart et al., 2001; Sandoz and Rockey, 2010; Schautteet and Vanrompay, 2011; Borel et al., 2016; Joseph et al., 2016; Seth-Smith et al., 2017). This gene is located within a genomic island carrying a class C tetracycline resistance gene and can be transferred to other *C. suis* but also to and among *Chlamydia trachomatis* upon simultaneous co-infection *in vitro* (Suchland et al., 2009; Jeffrey et al., 2013; Marti et al., 2017). This could have serious implications for human health considering that the DNA of both *C. suis* and *C. trachomatis* has been detected in the eye of trachoma patients in Nepal, and that *C. suis* could be isolated from samples of pig farmers and slaughterhouse workers originating from various anatomical locations (conjunctiva, nose, pharynx, and stool samples) (Dean et al., 2013; De Puysseleyr et al., 2014, 2015). Apart from tetracycline resistance in *C. suis*, induced resistance to antibiotics (e.g., rifamycins, fluoroquinolones) through point mutations has also been reported in *C. suis* and other *Chlamydia* (e.g., *C. trachomatis*, *C. pneumoniae*, *C. psittaci*, *C. muridarum*, and *C. caviae*) following propagation at sub-inhibitory concentrations of the drug *in vitro* (Sandoz and Rockey, 2010). Moreover, heterotypic resistance in various *Chlamydia* species has been observed upon survival of a small proportion of bacteria exposed to antibiotic concentrations well above the minimal inhibitory concentration (MIC) and might be a cause of treatment failure in patients, a phenomenon also referred to as drug indifference, tolerance, or persistence (Suchland et al., 2003; Sandoz and Rockey, 2010). In *Chlamydia*, persistence, or the chlamydial stress response, is known to occur specifically upon exposure to antibiotics affecting cell wall synthesis, such as penicillin (Lewis, 2007; Sandoz and Rockey, 2010; Schoborg, 2011; Borel et al., 2014; Zheng et al., 2015; Leonard et al., 2017; Xue et al., 2017).

In the present study, the MIC was determined according to the methods described by Donati et al. (2010) (MIC ≥ 90% inclusion number reduction) and by Suchland et al. (2003) [transition point MIC (MIC_TP_) ≥ 90% alteration of size/morphology; MIC = 2× MIC_TP_]. Following the determination of MIC (Donati) and MIC (Suchland), an MIC consensus was established if they were identical. Otherwise, an MIC range was determined unless the MICs were notably different. In addition, instead of applying the generally used protocols to determine the minimal bactericidal/chlamydicidal/lethal concentration (MBC/MCC/MLC), we incorporated an assay based on protocols used to evaluate recovery from the chlamydial stress response (Kintner et al., 2014; Leonard et al., 2015, 2017). With this recovery assay, it is possible to evaluate the *in vitro* behavior of chlamydial strains upon recovery and continued exposure to low, moderate, and high concentrations of the antibiotic in question instead of evaluating a single value that is identical to or only a few twofold dilutions higher compared to the determined MIC value as often observed for MBC/MCC/MLC (Henning and Krauss, 1986; Webberley et al., 1987; Ehret and Judson, 1988; Japan Society of Chemotherapy, 1992a,b; Agacfidan et al., 1993; Suchland et al., 2003; Donati et al., 2010).

**FIGURE 1:**
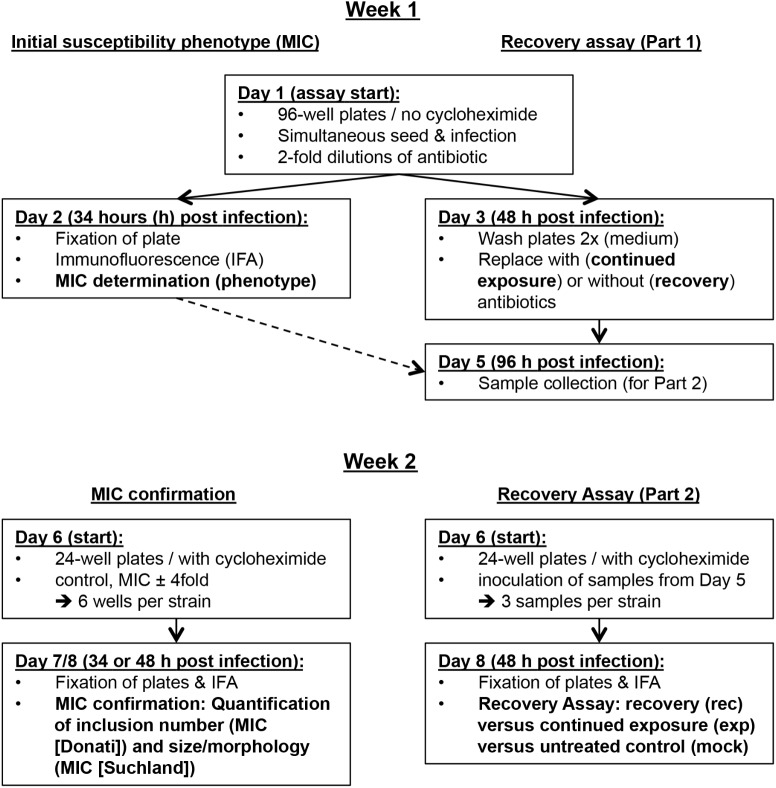
Overview of the *in vitro* antibiotic susceptibility assay protocol. Shown is an overview of the *in vitro* assay protocol grouped into the first and second week of the assay and divided into initial susceptibility phenotype/MIC confirmation and the recovery assay.

In summary, we propose a two-step protocol evaluating two major parameters, the MIC and the recovery. This approach allows initial assessment after two workdays, and a detailed report after eight workdays (**Figure 1**). For proof of principle, we investigated the susceptibility of three *C. suis* field strains and the reference strain S45/6 to tetracycline, sulfamethoxazole, and penicillin.

## Materials and Methods

The detailed *in vitro* antibiotic susceptibility assay protocol (tetracycline, sulfamethoxazole, and PenG) is available as Supplementary Text S1 and includes suggestions regarding optimization of this assay.

### Host Cells and Media

LLC-MK2 cells (Rhesus monkey kidney cell line, provided by IZSLER, Brescia, Italy) were grown in antibiotic-free growth medium consisting of 500 ml Eagle’s minimum essential medium (EMEM, Gibco, Thermo Fisher Scientific, Invitrogen, Carlsbad, CA, United States) supplemented with 10% heat-inactivated fetal calf serum (FCS, BioConcept, Allschwil, Switzerland), 5 ml L-glutamine (Gibco, Thermo Fisher Scientific), and 6 ml glucose (0.06 g/ml; Sigma-Aldrich Co., St. Louis, MO, United States). Following infection, growth medium was replaced by *Chlamydia* cultivation medium consisting of 500 ml EMEM supplemented with 20% FCS (BioConcept), 5 ml L-glutamine (Gibco, Thermo Fisher Scientific), and 2 g glucose (Sigma-Aldrich) with or without 0.7 ml cycloheximide (1 mg/ml; Sigma-Aldrich) as described (Donati et al., 2010; Wanninger et al., 2016).

### Chlamydial Strains

Four *C. suis* strains were used for this study: three field strains (SWA-14, 86, and 141), all isolated from fecal swabs of asymptomatic fattening pigs (Hoffmann et al., 2015; Wanninger et al., 2016) and the *C. suis* reference strain S45/6, which was originally isolated from feces of an asymptomatic pig (Kaltenboeck et al., 1993; Schautteet and Vanrompay, 2011; **Table 2**). Stocks were prepared and stored in sucrose phosphate glutamate (SPG) at -80°C [218 mM sucrose (Sigma-Aldrich), 3.76 mM KH_2_PO_4_ (Sigma-Aldrich), 7.1 mM K_2_HPO_4_ (Merck Eurolab AG, Dietlikon, Switzerland), and 5 mM GlutaMAX-100 (GIBCO)] as described (Leonard et al., 2016) with minor changes: After infected cells were scraped, mechanical disruption was performed by vortexing with 10–20 glass beads (∅ 5 mm) for 1 min followed by pushing the suspension through a 20 gauge needle with a 20 ml syringe.

**Table 2:** Overview of *Chlamydia suis* strains used in this study.

Sample ID	Year of isolation, country of origin	Antibiotic treatment of pig	European nucleotide archive deposition	References
SWA-14 *(alt. 2-7 a R)*	2013, Switzerland	Unknown	PRJEB17986	This study
SWA-86	2013, Switzerland	Unknown	PRJEB17986	This study
SWA-141 *(alt. 4-29 b R)*	2013, Switzerland	Tetracycline	PRJEB17986	Wanninger et al., 2016; Seth-Smith et al., 2017
S45/6 (ref)	1960s, Austria	Unknown	SRP076849	Kaltenboeck et al., 1993; Joseph et al., 2016

### Antibiotic Reagents

Tetracycline hydrochloride powder (Sigma-Aldrich) was dissolved in deionized water to reach a final concentration of 10 mg/ml, filter-sterilized with a 0.22 μm syringe filter, aliquoted, and stored at -20°C. Sulfamethoxazole (Santa Cruz Biotechnology, Dallas, TX, United States) stocks were prepared in DMSO (Sigma-Aldrich) with a final concentration of 50 mg/ml, filter-sterilized, and stored as described for tetracycline. PenG (sodium salt, Sigma-Aldrich) stocks were prepared as described (Leonard et al., 2016) with a stock concentration of 20,000 U/ml in deionized water.

### Important Definitions and Parameters

Minimal inhibitory concentration is defined as the consensus of the MIC defined by Donati et al. (2010; MIC Donati ≥ 90% inclusion number reduction) and Suchland et al. (2003; MIC Suchland ≥ 90% inclusions altered in size and morphology) unless one parameter could not be employed. In the recovery assay, chlamydial recovery upon drug-removal (rec) at 48 h is compared to both, continued exposure for 96 h (exp), and an untreated control (mock) following determination of the number of inclusion forming units (IFU)/ml (semi-quantitative analysis) and is expressed through three parameters. (1) The *resistance potential* represents how well *Chlamydia* resist continuous exposure to the antibiotic in question (exposure to mock). In detail, it provides the highest antibiotic concentration where (a) >25% or (b) >10% of continuously infected cultures remain infectious compared to untreated controls. (2) The *recovery potential* represents how well *Chlamydia* recover from antibiotic treatment (recovery to mock). It provides the highest antibiotic concentration where (a) >1% or (b) >10% of cultures remain infectious following recovery compared to the mock-exposed cultures. Finally, (3) *Survival after continued exposure* directly compares the infectivity of continuously exposed to recovered cultures (exposure to recovery) and provides the highest antibiotic concentration where (a) >1% or (b) >10% of continuously infected cultures remain infectious compared to the recovery data of the same antibiotic concentration.

### Overview of the *in Vitro* Antibiotic Susceptibility Assay

The protocol can be performed within 2 weeks and consists of (a) determination of the initial susceptibility phenotype, (b) MIC confirmation, and (c) the recovery assay. In the first week, the initial susceptibility phenotype and the first part of the recovery assay can be performed within 48 and 96 h, respectively. In the second week, MIC confirmation and the second part of the recovery assay are performed within two workdays. Because all samples required for the second week are stored frozen by the end of the first week, this provides a convenient potential stopping point in the protocol. Immunofluorescence microscopy analysis can be performed on the third day (**Figure 1**). For tetracycline, there is the possibility to initially perform a tetracycline resistance screen (Tet screen) for a large number of strains within a short period of time with minimal use of consumables.

### Determination of the Initial Susceptibility Phenotype

A *Chlamydia*/cell suspension was prepared for each *C. suis* strain containing 300,000 cells per ml cycloheximide-free *Chlamydia* cultivation medium and a multiplicity of infection (MOI) of 0.5. One hundred microliter per well of this suspension was added directly to 100 μl of cycloheximide-free *Chlamydia* cultivation medium containing a serial dilution of antibiotics in a 96-well plate (30,000 cells per well). For tetracycline, the final antibiotic concentrations ranged from 0.0078 to 8 μg/ml (twofold dilution); for sulfamethoxazole (or other antibiotics with an unknown susceptibility range), concentrations ranged from 4.9E-4 to 1024 μg/ml (twofold dilution); for PenG (and presumably appropriate for most or all known inducers of the classic chlamydial stress response), the final concentrations ranged from 0.01 to 100 U/ml in a 10-fold dilution series. For all antibiotics evaluated, an unexposed control (mock) containing only cycloheximide-free medium was added. Moreover, each strain was assayed in duplicate. Following centrifugation (1 h, 1000 *g*, 25°C), cultures were incubated at 37°C with 5% CO_2_ for 34 h before they were fixed in -20°C-chilled methanol for 10 min and immunolabelled according to the protocol described below. MIC determination was performed according to MIC (Donati). If an estimate regarding inclusion number reduction was not possible (e.g., sulfamethoxazole), MIC (Suchland) was employed instead.

### Recovery Assay Protocol

Cultures were performed identically to the initial susceptibility phenotype. After incubation for 48 h instead of 34 h, culture supernatants were removed, and monolayers were washed twice with antibiotic- and cycloheximide-free medium before addition of medium (150 μl) either without antibiotics (recovery) or with serially diluted antibiotics (continued exposure). After another incubation period (96 h post infection), samples were scraped into the supernatant and frozen at -80°C until use. For tetracycline, 0.03, 0.5, 2 μg/ml exposed samples and the unexposed mock control were collected both for recovery and continued exposure groups. Alternatively, 0.125 instead of 0.03 μg/ml could be collected. For sulfamethoxazole, 2, 32, 512 μg/ml exposed samples and the unexposed mock control were similarly collected. For penicillin, 1, 10, 100 U/ml exposed samples and the unexposed mock control were similarly collected. In total, each strain resulted in eight samples for IFU/ml determination per antibiotic agent (including mock). For antibiotics being evaluated for the first time, the general suggestion is that samples should be generated close to the lowest and the highest initially determined MIC, as well as including an intermediate concentration in order to evaluate the entire susceptibility range.

In the second week, samples were inoculated in duplicate onto 24-well plates containing coverslips of cells cultured to confluence. Following centrifugation (1 h, 1000 *g*, 25°C), inocula were replaced by 1 ml cycloheximide-containing *Chlamydia* cultivation medium. Monolayers inoculated with samples from the continuously exposed condition (including mock) were additionally washed twice with medium to remove residual drugs. Inoculation volumes were established for each antibiotic and generally consisted of 1 μl of 1 ml total sample volume for the mock controls (details shown in Supplementary Text S1). For resistant strains (determined according to the initial susceptibility phenotype) 1, 10, and 60 μl were generally inoculated for low, intermediate, and high concentrations, respectively. For sensitive strains (according to the initially determined phenotype), 1 or 10 μl inocula were used for samples, which exhibited recovery from low antibiotic concentrations (MIC or below) and 60 μl for all other conditions/samples. Following fixation in methanol and immunofluorescence assay (IFA), the IFU/ml for each condition (recovery, or continued exposure for mock and antibiotic concentrations) was determined according to previously published methods (Deka et al., 2006). (a) The resistance potential, (b) the recovery potential, and (c) survival after continued exposure was determined as described above (“Important definitions and parameters”).

### MIC Confirmation

Minimal inhibitory concentration confirmation was performed in the second week. Prepared host cell monolayers on glass coverslips in 24-well plates (150,000 cells per well) were infected with an MOI of 0.1 in 1 ml cycloheximide-containing *Chlamydia* cultivation medium. Following centrifugation (1 h, 1000 *g*, 25°C), inocula were replaced with 1 ml cycloheximide-containing *Chlamydia* cultivation medium either without (mock) or with antibiotics close to the MIC. For tetracycline, 0.03, 0.125, and 0.5 μg/ml were used; for sulfamethoxazole, 64, 128, and 256 μg/ml were used for the field strains, and for S45/6, in addition to these concentrations, 0.0039, 0.0078, and 0.015 μg/ml were tested; for PenG, 1, 10, and 100 U/ml was used. Following incubation for 34 h (tetracycline) or 48 h (sulfamethoxazole, penicillin), monolayers were fixed in methanol and IFA was performed. MIC determination was performed according to definitions by Suchland et al. (2003) and Donati et al. (2010) as described above.

In detail, the average number of inclusions per 20× field was determined and compared to the mock for MIC (Donati). The lowest antibiotic concentration, where 10% or less inclusions were present compared to the mock, was defined as the MIC.

For MIC (Suchland), the MIC was based on two different criteria: inclusion size and morphology, neither of which can be as easily quantified as the inclusion number due to possible variability. Despite this drawback, MIC determination was possible as the change from normal to altered inclusions in 90% of the inclusions was abrupt rather than gradual. In order to quantify this change, we semi-quantitatively determined the mean inclusion size. For that, 50 randomly selected inclusions were evaluated in at least 10 fields (400× magnifications) per condition, and the area (in μm^2^) was calculated using BonTec measuring and archiving software (BonTec, Bonn, Germany). Representative microscopic images were captured using BonTec software (BonTec) and a UI-2250SE-C-HQ camera (uEye, IDS Imaging Development Systems GmbH, Obersulm, Germany) as described previously (Leonard et al., 2015). For conditions with only few or very small inclusions, up to 20 inclusions were analyzed for size and morphology if possible. In parallel, we qualitatively evaluated inclusion morphology. The following criteria were used to classify the inclusion morphology as altered compared to control inclusion morphology: size and/or the presence of aberrant bodies (ABs), aberrant inclusion bodies; diameter ≥ 2 μm (Matsumoto and Manire, 1970). Micro-inclusions were defined as inclusions with an area of less than 15 μm^2^. In the case of discrepancies between these parameters, we reported susceptibility ranges (e.g., 64–128 μg/ml).

### Tetracycline Resistance Screen (Tet Screen)

With this method, up to 10 strains per 24-well plate can be tested for tetracycline susceptibility. A tetracycline sensitive and a resistant control should be included. Two wells of a 24-well plate with confluent monolayers (150,000 cells/well) on glass coverslips are infected with an MOI of approximately 0.5 for each strain. Following centrifugation (1 h, 1000 *g*, 25°C), inocula are replaced with 1 ml of cycloheximide-containing medium either with or without 0.125 or 0.5 μg/ml tetracycline. After 34 h of incubation, monolayers are fixed in methanol, immunolabelled, and tested for the presence or absence of inclusions in tetracycline-containing conditions. If inclusions in tetracycline-exposed cultures are comparable to the corresponding unexposed-control, in terms of inclusion number and morphology, the strain is considered tetracycline resistant. Complete absence of inclusions upon tetracycline exposure indicates that the strain is tetracycline sensitive. Strains falling in between these categories (“intermediate”) should be further analyzed with the *in vitro* susceptibility assay protocol described above.

### Immunofluorescence Analysis (IFA)

Inclusions and cell nuclei were visualized as described previously (Leonard et al., 2016). Briefly, primary *Chlamydiaceae* family-specific mouse monoclonal antibody directed against the chlamydial lipopolysaccharide (LPS, Clone ACI-P; Progen, Heidelberg, Germany; 1:200) and secondary Alexa Fluor 488-conjugated secondary goat anti-mouse antibody (Molecular Probes, Eugene, OR, United States; 1:500) were used to label inclusions. Host and chlamydial DNA were labelled with 1 μg/ml 4′,6-diamidino-2-phenylindole dihydrochloride (DAPI, Molecular Probes). If applicable (for MIC confirmation and recovery assay), coverslips were mounted with FluoreGuard mounting medium (Hard Set; ScyTek Laboratories Inc., Logan, UT, United States) on glass slides. All coverslips were analyzed with a Leica DMLB fluorescence microscope (Leica Microsystems, Wetzlar, Germany), while 96-well plates were analyzed with a Nikon Eclipse T*i* microscope (Nikon, Tokyo, Japan).

### Transmission Electron Microscopy (TEM)

Transmission electron microscopy (TEM) images were produced according to previously published methods (Leonard et al., 2016). Ultrathin (80 nm) sections were mounted on gold grids (Merck) and contrasted with uranyl acetate dehydrate (Fluka; Sigma-Aldrich) and lead citrate (Merck). Sections were subsequently evaluated using a Philips CM10 electron microscope (Software release version 5.1; FEI Company, Hillsboro, OR, United States) and imaged using a Gatan Orius SC1000 CCD Camera with software version Digital Micrograph 2.30 (Gatan Inc., Warrendale, PA, United States).

### Statistical Analysis

Unless stated otherwise, results were displayed as means ± standard deviation, of the results from two or three independent experiments. Statistical significance of the difference of means was determined by Student’s *t*-test and Welch *t*-test (*t*-test unequal variance) using GraphPad QuickCalcs Website^1^ and the Excel software. *p*-values of <0.05 were considered significant.

## Results

### Tetracycline Susceptibility Can Be Determined Using Multiple Evaluation Parameters

#### Initial Susceptibility Phenotype and MIC Determination

The initial tetracycline susceptibility phenotype of four *C. suis* strains (field strains SWA-14, SWA-86, and SWA-141, and reference strain S45/6), all originally isolated from farmed pigs, was determined after two to three workdays. This was accomplished by simultaneously seeding and infecting LLC-MK2 cells with *Chlamydia* in cycloheximide-free medium with twofold tetracycline dilutions (Week 1, **Table 3**) and determining the MIC as described by Donati et al. (2010; **Table 3**). In confirmation of previously published results (Dugan et al., 2004), and in accord with tetracycline susceptibility definitions described (Wanninger et al., 2016), S45/6 was sensitive to tetracycline (MIC < 2 μg/ml). Among the field strains, SWA-141 was resistant (MIC ≥ 4 μg/ml), and SWA-14 and SWA-86 were sensitive, and no strain showed intermediate tetracycline susceptibility (2 μg/ml ≤ MIC < 4 μg/ml) (Dugan et al., 2004; Wanninger et al., 2016). The MIC was confirmed by infecting confluent host cell monolayers in the presence of tetracycline concentrations several twofold dilutions above and below the initially determined MIC in cycloheximide-containing medium (Week 2, **Table 3**). MIC determination was performed both according to Suchland et al. (2003) and Donati et al. (2010) to establish the MIC consensus. Cycloheximide was not added in the first week to ensure cellular growth upon simultaneous seed and infection, while it was added in the second week to (a) analyze whether cycloheximide potentially influences the susceptibility of *Chlamydia* to tetracycline and other antibiotics and (b) to avoid overgrowth of the cell monolayers.

**Table 3:** Summary of the MIC determination (tetracycline).

	SWA-14	SWA-86	SWA-141	S45/6
**Week 1**				
MIC (per Donati)	0.03 μg/ml	0.03 μg/ml	4 μg/ml	0.06 μg/ml
**Week 2**				
MIC (per Donati)	0.06 μg/ml	0.03 μg/ml	4 μg/ml	0.125 μg/ml
MIC (per Suchland)	0.06 μg/ml	0.03 μg/ml	4 μg/ml	0.06–0.125 μg/ml
**MIC consensus**	**0.03–0.06 μg/ml**	**0.03 μg/ml**	**4 μg/ml**	**0.06–0.125 μg/ml**
***Interpretation***	***Sensitive***	***Sensitive***	***Resistant***	***Sensitive***

#### Inclusion Size Analysis

**FIGURE 2:**
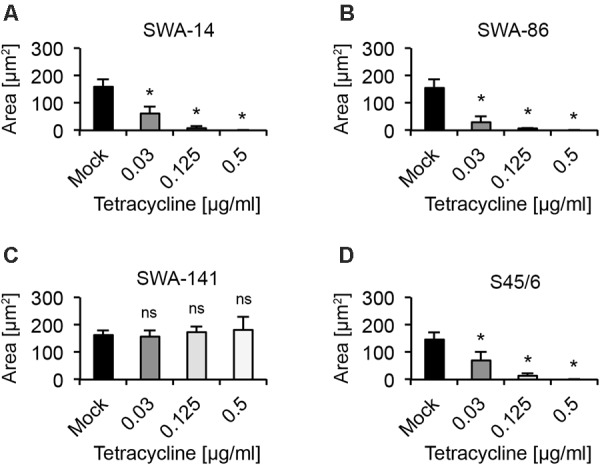
Average inclusion size analysis upon tetracycline exposure. The bar graphs compare the average inclusion size (μm^2^) of unexposed controls (mock) with tetracycline-exposed (0.03, 0.125, and 0.5 μg/ml) cultures at 34 h post infection (mean ± SD). Shown are the results for strains **(A)** SWA-14, **(B)** SWA-86, **(C)** SWA-141, and **(D)** reference strain S45/6. Asterisks indicate a statistically significant difference between the tetracycline-exposed cultures and the mock by both Student’s *t*-test and the Welch *t*-test (ns, not significant; ^∗^*p* < 0.05; ^∗∗^*p* < 0.01). Three independent experiments were performed (*n* = 3); 0.125 and 0.5 μg/ml tetracycline were only evaluated in two of the three experiments (*n* = 2).

Considering data provided by Suchland et al. (2003), a reduction in inclusion numbers can be expected to be preceded or accompanied by altered inclusion size and/or morphology. In order to quantify and further confirm the MIC (Suchland), we analyzed the average inclusion size for each strain at 0.03, 0.125, and 0.5 μg/ml tetracycline. We clearly observed that the average inclusion size of all three tetracycline sensitive strains (SWA-14, SWA-86, S45/6) was significantly reduced at tetracycline concentrations as low as 0.03 μg/ml, while the average inclusion size of tetracycline resistant SWA-141 exposed to ≤ 0.5 μg/ml tetracycline was similar to the unexposed control (mock, **Figure 2**).

#### Tetracycline Resistance Screen (Tet Screen)

**FIGURE 3:**
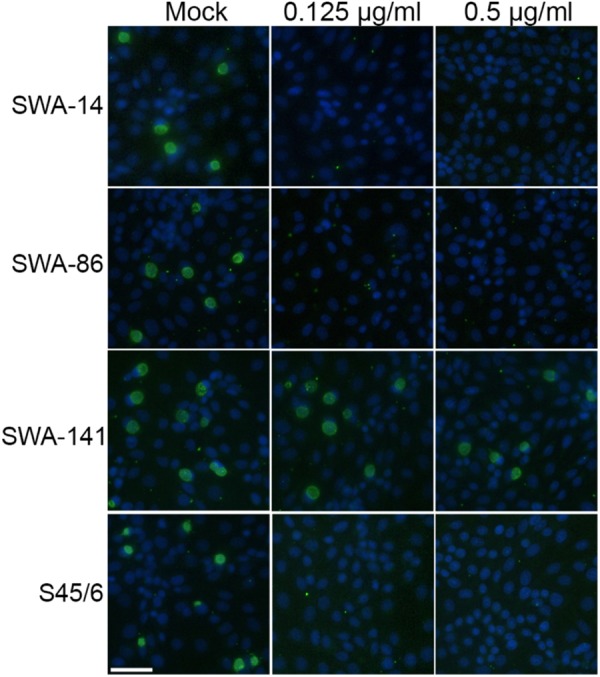
Tetracycline resistance screen of strains SWA-14, 86, 141, and S45/6. Shown are immunofluorescence images illustrating the results of the tetracycline resistance screen (TET Screen) wherein inclusion number and morphology of mock-exposed chlamydial strains are compared to cultures exposed to 0.125 or 0.5 μg/ml tetracycline. The white bar represents 50 μm.

The observed variation in inclusion size in tetracycline resistant versus sensitive strains led us to hypothesize that it might be possible to easily distinguish resistant from sensitive strains by comparing the unexposed controls to cultures exposed to 0.125 or 0.5 μg/ml tetracycline, which would allow screening of a large number of strains in less than 48 h post infection (hpi). **Figure 3** shows that the inclusion number and size/morphology of the tetracycline resistant strain SWA-141 was similar regardless of tetracycline concentration, while no or only micro-inclusions (area less than 15 μm^2^) were visible in tetracycline-exposed cultures infected with tetracycline sensitive SWA-14, 86, or S45/6. To further confirm our hypothesis, we screened additional previously reported tetracycline resistant (5–27b, 1–28b, 5–22b) and sensitive (10–26b, 1–28a) *C. suis* field strains from previous studies (Wanninger et al., 2016; Seth-Smith et al., 2017). As expected, we also observed that inclusion number and morphology of the three tetracycline resistant strain was not affected by tetracycline exposure while the two sensitive strains showed no or only micro-inclusions at 0.125 and 0.5 μg/ml tetracycline (Supplementary Figure S1). In addition, we screened two human chlamydial strains, *C. trachomatis* serovar E and *C. pneumoniae* Kajaani 6, and confirmed their susceptibility to tetracycline, as indicated by the described inclusion size criteria, as expected (Supplementary Figure S2).

#### Recovery Assay

Finally, we analyzed the infectivity of strains SWA-14, 86, 141, and S45/6, in terms of recovery, upon exposure to tetracycline followed by discontinuation of exposure to tetracycline versus uninterrupted, continuous exposure to tetracycline. Briefly, in parallel to the initial susceptibility phenotype, cultures were prepared for the recovery assay in cycloheximide-free medium. However, instead of fixation of the infected cells on coverslips, followed by immunofluorescence microscopic analysis to determine the phenotype, infected cells were washed two times with fresh medium, to remove residual drugs, and the medium was replaced with either medium alone (recovery, rec) or the same concentrations of tetracycline (continued exposure, exp) and incubated for another 48 h prior to scraping collection of infected cells to determine infectivity. In week 2, in parallel to performing MIC confirmation, collected samples were inoculated onto fresh cells in cycloheximide-containing medium to determine the IFU/ml (see the Materials and Methods section). The complete, detailed protocol is available in Supplementary Text S1.

**FIGURE 4:**
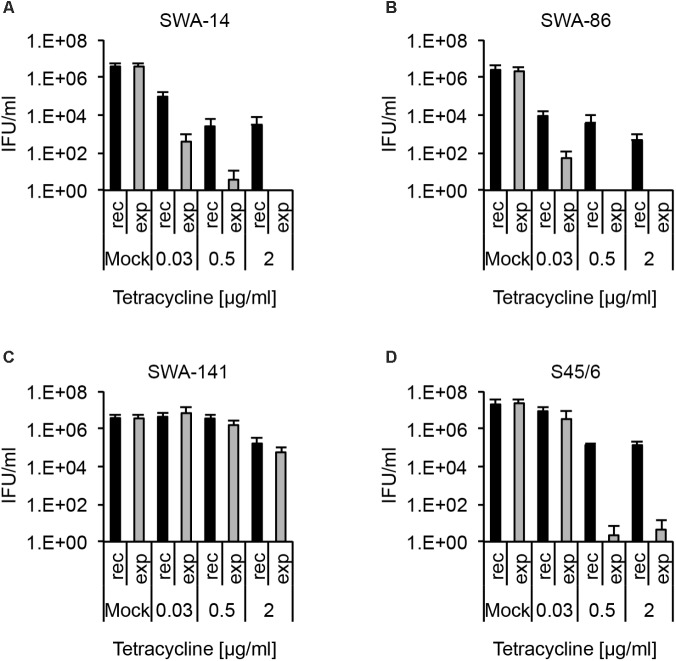
Recovery assay following chlamydial infection and tetracycline exposure. The bar graphs compare the average inclusion forming units per ml (IFU/ml) of unexposed controls (mock) with tetracycline-exposed (0.03, 0.5, or 2 μg/ml) chlamydial cultures (mean ± SD). Cultures were either continuously exposed (exp) to tetracycline for 96 h or exposed to tetracycline for 48 h, then further cultured in tetracycline-free medium for 48 h (recovery, rec). Shown are the results for strains **(A)** SWA-14, **(B)** SWA-86, **(C)** SWA-141, and **(D)** reference strain S45/6. Three independent experiments were performed (*n* = 3).

We found that even tetracycline sensitive strains (SWA-14, 86, S45/6) are able to recover from tetracycline concentrations as high as 2 μg/ml, despite MIC values of 0.125 μg/ml or less (**Figure 4**). In contrast, no or only a minimal number of IFU (<0.001% of mock) were detectable following continued exposure to 0.5 and 2 μg/ml tetracycline for SWA-14, SWA-86, and S45/6, while the infectivity of SWA-141 cultures continuously exposed to 0.5 and 2 μg/ml was equivalent to 43.96% and 1.56% of the mock exposed infection, respectively.

In order to concisely summarize the data (here for tetracycline exposure, also useful for analyses of other antibiotics) and provide descriptive characterization of the strains, we developed the following data analyses for the data (see **Table 4**): (1) “Resistance potential” (compares exposure to mock groups and represents the degree to which *Chlamydia* resist continuous exposure to the antibiotic in question) indicates the highest tetracycline concentration at which tetracycline exposed cultures exhibit infectivity equivalent to (a) >25% or (b) >10% of mock-exposed culture infectivity. (2) “Recovery potential” (compares recovery to mock groups and represents the degree to which *Chlamydia* recover from antibiotic exposure) indicates the highest antibiotic concentration at which cultures initially exposed to tetracycline, but then further cultured in the absence of tetracycline (recovered cultures), exhibit infectivity equivalent to (a) >1% or (b) >10% of mock-exposed culture infectivity. Lastly, (3) “Survival after continued exposure” [directly compares the infectivity of continuously tetracycline exposed cultures to recovered cultures (exposed to recovered groups)] indicates the highest antibiotic concentration at which continuously exposed cultures exhibit infectivity equivalent to (a) >1% or (b) >10% of recovered culture infectivity. The result of these analyses allowed clear differentiation between tetracycline resistant (SWA-141) and sensitive (SWA-14, SWA-86, and S45/6) strains (**Table 4**).

**Table 4:** Summary of the recovery assay (tetracycline), strain characterization.

	SWA-14	SWA-86	SWA-141	S45/6
**(a) Resistance potential (exposure to mock)**		
>25%	<0.03 μg/ml	<0.03 μg/ml	0.5 μg/ml	<0.03 μg/ml
>10%	<0.03 μg/ml	<0.03 μg/ml	0.5 μg/ml	0.03 μg/ml
**(b) Recovery potential (recovery to mock)**		
>10%	<0.03 μg/ml	<0.03 μg/ml	0.5 μg/ml	0.03 μg/ml
>1%	0.03 μg/ml	<0.03 μg/ml	2 μg/ml	0.03 μg/ml
**(c) Survival post exposure (exposure to recovery)**		
>10%	<0.03 μg/ml	<0.03 μg/ml	2 μg/ml	0.03 μg/ml
>1%	<0.03 μg/ml	<0.03 μg/ml	2 μg/ml	0.03 μg/ml
*Interpretation*	*Sensitive*	*Sensitive*	*Resistant*	*Sensitive*

### Reference Strain S45/6 Is Sensitive to Sulfamethoxazole, While All Tested *C. suis* Field Strains Are Sulfamethoxazole Resistant

#### Initial Susceptibility Phenotype, Inclusion Size Analysis and MIC Determination

As for tetracycline, we aimed to analyze the susceptibility of the same four *C. suis* strains to sulfamethoxazole. However, determination of the initial susceptibility phenotype according to Donati et al. (2010) was not possible due to the fact that we only observed sulfamethoxazole-dependent changes in inclusion size/morphology, but no sulfamethoxazole-dependent changes in the number of inclusions, even at concentrations as high as 512 μg/ml sulfamethoxazole compared to the mock-exposed control (Supplementary Figure S3A). As a result, initial susceptibility phenotype determination was performed only according to Suchland et al. (2003), resulting in MIC of 128–256, 64–128, 128–256, and 0.0039 μg/ml for SWA-14, 86, 141, and S45/6, respectively.

**FIGURE 5:**
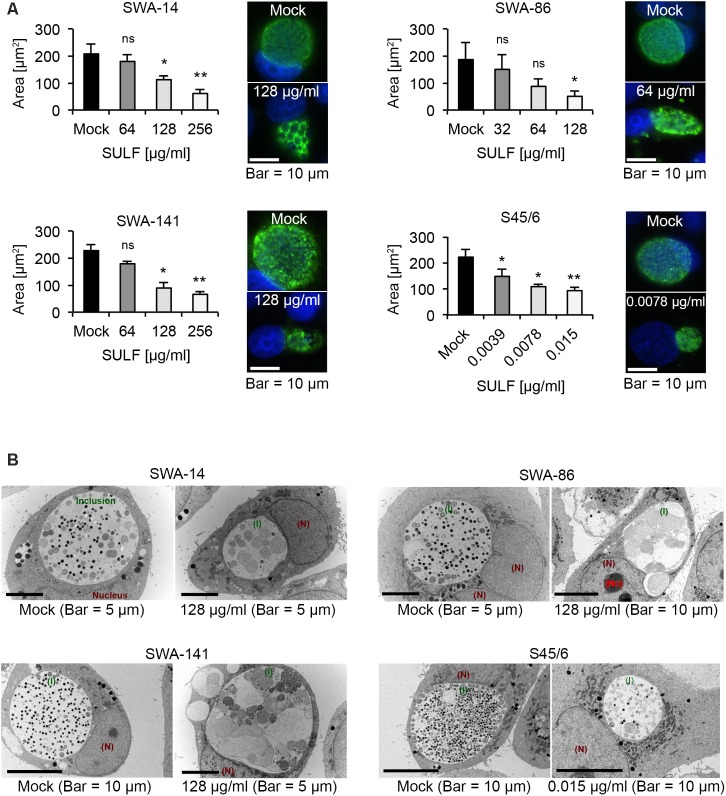
The effects of sulfamethoxazole exposure. **(A)** Shown are bar graphs comparing the average inclusion size of sulfamethoxazole-treated cultures to the mock-exposed cultures (left; mean ± SD) as well as representative immunofluorescence images of the inclusion morphology of the untreated control (right, top) and the MIC_TP_ (right, bottom) for strains SWA-14, SWA-86, SWA-141, and S45/6 at 48 h post infection. Asterisks indicate a statistically significant difference between the sulfamethoxazole-exposed cultures and the mock by both Student’s *t*-test and the Welch *t*-test (ns, not significant; ^∗^*p* < 0.05; ^∗∗^*p* < 0.01). Three independent experiments were performed (*n* = 3). **(B)** Representative transmission electron microscopy (TEM) images are shown for each strain wherein the mock is presented on the left part of the panel and sulfamethoxazole-exposed cultures are presented on the right. Inclusions are indicated in green (I), the nucleus in dark red (N) and, if applicable, the nucleolus in bright red (Nc). Cultures were fixed at 48 h post-infection for processing for TEM analysis.

To confirm these MIC, we evaluated the average inclusion size per strain at sulfamethoxazole concentrations close to the initially determined MIC as performed for tetracycline. **Figure 5A** illustrates the inclusion size differences allowing initial determination of MIC (per Suchland) and subsequent confirmation. The entire data set is summarized in **Table 5**.

**Table 5:** Summary of the MIC determination (sulfamethoxazole).

	SWA-14	SWA-86	SWA-141	S45/6
Initially determined MIC	128–256 μg/ml	64–128 μg/ml	128–256 μg/ml	0.0039 μg/ml
MIC confirmation	256 μg/ml	64–128 μg/ml	128–256 μg/ml	0.0078 μg/ml
**MIC consensus**	**128–256 μg/ml**	**64–128 μg/ml**	**128–256 μg/ml**	**0.0039–0.0078 μg/ml**
***Interpretation***	***Resistant***	***Resistant***	***Resistant***	***Sensitive***

Interestingly, upon MIC confirmation, we observed that the three field strains exposed to sulfamethoxazole at their respective MIC (ranging from 64 to 256 μg/ml) universally contained smaller inclusions by IFA compared to the mock-exposed infection. Those inclusions appeared to contain large ABs indicative of the chlamydial stress response. In contrast, while a significant inclusion size reduction was apparent upon exposure to 0.0039 μg/ml sulfamethoxazole for reference strain S45/6, ABs were only sporadically present in the reference strain (**Figure 5A**).

#### Transmission Electron Microscopy Analysis of Sulfamethoxazole-Exposed Cultures

To confirm IFA observations regarding the presence of ABs, we performed TEM analysis (**Figure 5B**). ABs were defined as pale, large inclusions of round to irregular shape with ≥2 μm in diameter (Matsumoto and Manire, 1970). While all mock-exposed cultures had inclusions primarily populated with small elementary bodies (EBs; dark, 0.25–0.5 μm in diameter) and reticulate bodies (RBs; pale, 0.5–1 μm in diameter), field strain cultures exposed to 128 μg/ml sulfamethoxazole contained inclusions populated with a large number of ABs or markedly altered RBs. In contrast, S45/6 cultures exposed to 0.015 μg/ml sulfamethoxazole contained a decreased number of EBs and RBs but no obvious increase in the number of ABs, confirming IFA results.

#### Recovery Assay

Finally, we analyzed the infectivity of strains SWA-14, SWA-86, SWA-141, and S45/6 in terms of recovery upon exposure to sulfamethoxazole followed by discontinuation of exposure to sulfamethoxazole versus continuous exposure to sulfamethoxazole. The assay was performed identically to the described tetracycline recovery assay.

**FIGURE 6:**
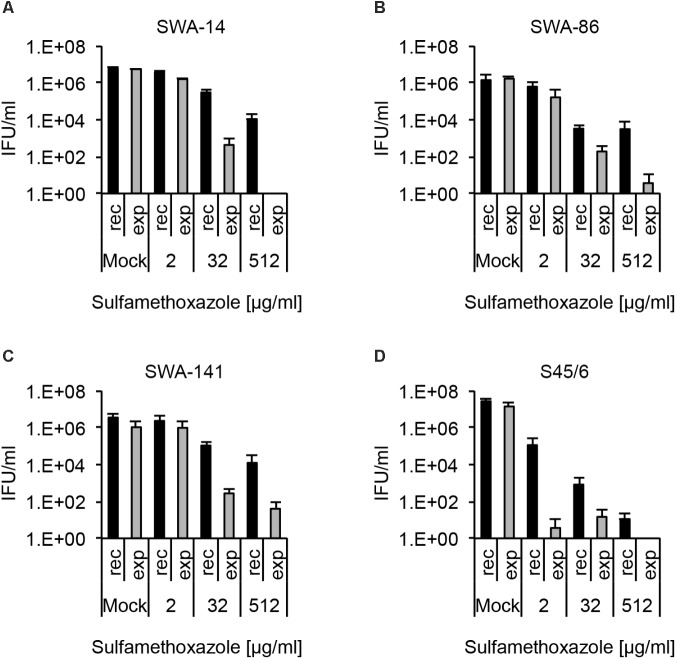
Recovery assay following chlamydial infection with sulfamethoxazole exposure. The bar graphs compare the average inclusion forming units per ml (IFU/ml) of unexposed controls (mock) with sulfamethoxazole-exposed (2, 32, and 512 μg/ml) cultures (mean ± SD). Cultures were either continuously exposed (exp) to sulfamethoxazole for 96 h or exposed to sulfamethoxazole for 48 h, then further cultured in sulfamethoxazole-free medium for 48 h (recovery, rec). Shown are the results for strains **(A)** SWA-14, **(B)** SWA-86, **(C)** SWA-141, and **(D)** reference strain S45/6. Three independent experiments were performed (*n* = 3).

As expected from the initial susceptibility phenotype, wherein no sulfamethoxazole-dependent inclusion number reduction was detected, all strains showed recovery up to 512 μg/ml sulfamethoxazole though the number of IFU was distinctly reduced to only 0.18, 0.22, 0.35, and <0.001% of the control for SWA-14, 86, 141, and S45/6, respectively. While the general recovery pattern, as well as survival following continuous sulfamethoxazole exposure, was comparable for the three field strains, S45/6 showed a relative reduction of both parameters compared to the field strains (**Figure 6**). This sulfamethoxazole susceptibility difference between the field strains and the type strain was even more pronounced when additional sulfamethoxazole concentrations were evaluated (2, 8, 32, 128, 512 μg/ml sulfamethoxazole for field strains and 0.0039, 0.03, 0.25, 2, 8, 32, 128, 512 μg/ml sulfamethoxazole for S45/6, Supplementary Figure S3B).

Again, as for tetracycline analysis above, we compiled a table containing the following data of sulfamethoxazole analyses: (1) “resistance potential” (compares exposure to mock groups), (2) “recovery potential” (compares recovery to mock groups), and (3) “survival after continued exposure” (compares exposure to recovery groups). The results of these analyses allowed clear differentiation between sulfamethoxazole resistant field strains (SWA-14, 86, 141) and the sulfamethoxazole sensitive reference strain S45/6 (**Table 6**).

**Table 6:** Summary of the recovery assay (sulfamethoxazole), strain characterization.

	SWA-14	SWA-86	SWA-141	S45/6
**(a) Resistance potential (exposure to mock)**		
>25%	<2 μg/ml	<2 μg/ml	2 μg/ml	<0.0039 μg/ml
>10%	2 μg/ml	2 μg/ml	2 μg/ml	<0.0039 μg/ml
**(b) Recovery potential (recovery to mock)**		
>10%	2 μg/ml	2 μg/ml	2 μg/ml	<0.0039 μg/ml
>1%	32 μg/ml	2 μg/ml	32 μg/ml	0.0039 μg/ml
**(c) Survival post exposure (exposure to recovery)**		
>10%	2 μg/ml	2 μg/ml	2 μg/ml	<0.0039 μg/ml
>1%	2 μg/ml	32 μg/ml	2 μg/ml	0.0039 μg/ml
*Interpretation*	*Resistant*	*Resistant*	*Resistant*	*Sensitive*

### Penicillin G (PenG) Induces the Chlamydial Stress Response in *C. suis* Strains

#### Initial Susceptibility Phenotype, Inclusion Size Analysis and MIC Determination

**FIGURE 7:**
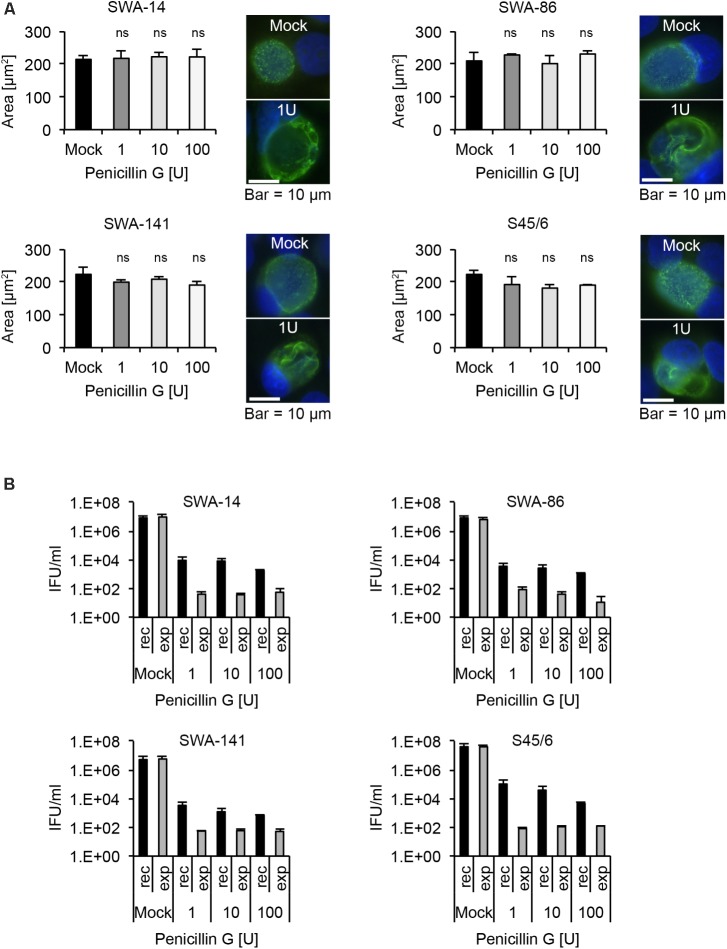
Penicillin G induces the chlamydial stress response in *all C. suis* strains. **(A)** Shown are bar graphs comparing the average chlamydial inclusion size of Penicillin G (PenG)-exposed cultures to the mock-exposed control (left; mean ± SD) as well as representative immunofluorescence microscopy images of inclusion morphology of both the unexposed control (right, top) and cultures exposed to 1 U/ml of PenG (right, bottom) for strains SWA-14, SWA-86, SWA-141, and S45/6 at 48 h post infection. Two independent experiments were performed (*n* = 2). Asterisks indicate a statistically significant difference between the PenG-exposed cultures and the mock by both Student’s *t*-test and the Welch *t*-test (ns, not significant; ^∗^*p* < 0.05; ^∗∗^*p* < 0.01). **(B)** The bar graphs compare the average inclusion forming units per ml (IFU/ml) of unexposed controls (mock) with PenG-exposed (1, 10, and 100 U/ml) cultures (mean ± SD). Cultures continuously exposed to PenG for 96 h (exp) were compared with cultures exposed to PenG for 48 h and then further cultured in PenG-free medium for 48 h (recovery, rec). Shown are the results for strains SWA-14 (top left), SWA-86 (top right), 141 (bottom left), and the reference strain S45/6 (bottom right). Two independent experiments were performed (*n* = 2).

PenG is known to induce the chlamydial stress response or persistence in chlamydial species such as *C. trachomatis* and *C. muridarum*, the closest phylogenetic relatives of *C. suis* (Phillips Campbell et al., 2012; Kintner et al., 2014; Joseph et al., 2016; Seth-Smith et al., 2017). In our study, we performed a PenG 10-fold serial dilution ranging from 0.001 to 100 U/ml PenG and observed that almost 100% of *C. suis* inclusions appeared to be altered in terms of morphology with the presence of ABs at almost all concentrations tested (0.1–100 U/ml for the field strains and 0.01–100 U/ml for S45/6, data not shown). At 0.001 U/ml PenG, less than 90% of inclusions were aberrant in all evaluated strains. We subsequently performed MIC determinations while evaluating inclusion size and morphology at PenG concentrations of 1, 10, and 100 U/ml. Interestingly, while the inclusion morphology clearly showed signs of persistence in all four strains (SWA-14, SWA-86, SWA-141, S45/6) even at the lowest PenG concentration of 1 U/ml, the average inclusion size remained similar to that of the mock control (**Figure 7A**). The observation of PenG-induced ABs in *C. suis* was confirmed by TEM comparing inclusions of S45/6 exposed to 1 and 100 U/ml PenG with the mock-exposed control. PenG-exposed *C. suis* inclusions consisted of mostly empty inclusions containing few ABs (Supplementary Figure S4A) as described recently for *C. trachomatis* and *C. pecorum* (Kintner et al., 2014; Leonard et al., 2016).

#### Recovery Assay

As a final step, we analyzed the infectivity of strains SWA-14, SWA-86, SWA-141, and S45/6 in terms of recovery upon exposure to PenG followed by discontinuation of exposure to PenG versus continuous exposure to PenG. Recovery assays were performed identically to the described tetracycline and sulfamethoxazole recovery assays. As expected, all the *C. suis* strains that were evaluated exhibited increased infectivity upon discontinuation of PenG exposure for 48 h, after 48 h of culture in the presence of PenG, compared to the continuous PenG-exposure group. Though the recovery group infectivity never exceeded 0.001% of the infectivity of the unexposed control (which was inoculated with *Chlamydia* and cultured for 48 h in the absence of PenG exposure), discontinuation of PenG exposure for 48 h resulted in infectivity levels 10- to 20-fold greater than those observed for cultures subjected to continuous PenG exposure for the same duration, indicative of recovery from PenG-induced infectivity reduction. Very little infectivity (<150 IFU/ml)/single inclusions were detected in the recovery assay for continuously exposed cultures for all PenG concentrations evaluated (**Figure 7B**). Again, TEM analysis was performed for S45/6 to further demonstrate recovery after removal of PenG. When PenG exposure was discontinued after 48 h of exposure and culture was continued in the absence of PenG for 48 h, inclusions contained normal EBs and RBs, while continuously exposed cultures continued to contain only few inclusion bodies primarily consisting of ABs (Supplementary Figure S4B).

## Discussion

While there are numerous standardized methods to evaluate extracellular and facultative intracellular bacteria regarding susceptibility to antibiotic agents, there are no standardized antibiotic susceptibility assays for *Chlamydia*. Instead, several research groups have, over time, developed multiple protocols to determine the antibiotic susceptibility of *Chlamydia in vitro* with microscopic methods as summarized in **Table 1** (Henning and Krauss, 1986; Webberley et al., 1987; Ehret and Judson, 1988; Japan Society of Chemotherapy, 1992a,b; Agacfidan et al., 1993; Suchland et al., 2003; Donati et al., 2010). In addition to these classic *in vitro* susceptibility protocols, there are other systems such as antibiotic susceptibility in continuous-infection models instead of regular infection *in vitro* (Kutlin et al., 1999), *in vitro susceptibility* testing using flow cytometry instead of classic determination via microscopic reading (Dessus-Babus et al., 1998), and the use of a reverse transcriptase PCR (RT-PCR)-based method instead of inclusion number determination (Cross et al., 1999).

Regardless of the applied method, a clear definition of the MIC and other evaluation parameters is needed to implement new approaches to determine the *in vitro* antibiotic susceptibility of *Chlamydia*. Although there has been no gold standard or consensus in the field for a standardized protocol for determination of antibiotic susceptibility and resistance, all research groups prior to 2000 defined the MIC as the lowest concentration where no inclusions were found (Hammerschlag, 1982; Henning and Krauss, 1986; Webberley et al., 1987; Ehret and Judson, 1988; Japan Society of Chemotherapy, 1992a,b; Agacfidan et al., 1993). More recent protocols from the 2000s included the principle of inclusion number reduction by 90% or more (Donati et al., 2010) or alterations in inclusion size and morphology (Suchland et al., 2003). Suchland et al. (2003) specifically noted that it might be problematic to define the MIC according to few survivors at high antibiotic concentrations (heterotypic survival), since micro-inclusions may not be visible depending on the staining/labeling method and magnification used. In view of these publications, we developed a consensus MIC considering previous MIC definitions according to Suchland et al. (2003) and Donati et al. (2010).

Unlike already published protocols to determine the MIC, which are analyzed differently but processed analogously, the method to determine MBC/MCC/MLC strongly depends on the research group (**Table 1**). In detail, while one half of all research groups defined the MBC/MCC/MLC as the lowest concentration where there were no inclusions after passaging the strain once in drug-free medium (Webberley et al., 1987; Ehret and Judson, 1988; Agacfidan et al., 1993; Suchland et al., 2003), the rest defined the MBC/MCC/MLC as the lowest antibiotic concentration where there were no or 90% fewer inclusions following re-incubation in drug-free medium compared to the mock-exposed control (Henning and Krauss, 1986; Japan Society of Chemotherapy, 1992a,b; Donati et al., 2010). Regardless of the protocol, all MBC/MCC/MLC protocols only determine one value, which is usually identical or only a few twofold dilutions higher compared to its MIC. In this study, in order to complement the MIC and to further characterize the chlamydial response to antibiotic exposure *in vitro*, we decided to employ the recovery assay, which has so far been described in studies investigating the chlamydial stress response (Kintner et al., 2014; Leonard et al., 2015, 2017). With this recovery assay, instead of determining one single value that gives little additional information to the MIC, we evaluated low, intermediate, and high antibiotic concentrations for effects upon chlamydial infectivity after discontinuation of antibiotic exposure 48 hpi (recovery) or continued exposure for 96 h. Not only does it allow us to confirm the impact of inclusion size reduction on subsequent infectivity, it further implements the MBC/MCC/MLC (recovery) and expands previous protocols to observing infectivity upon continued exposure.

In the present study, we evaluated the antibiotic susceptibility of three porcine field strains in comparison to the *C. suis* reference strain S45/6 (also of porcine origin) to tetracycline, sulfamethoxazole, and penicillin. These antibiotic agents were chosen because of their extensive use in the pig farming industry (Hoffmann et al., 2015). Moreover, the presence of the tetracycline resistance gene *tet*A(C) has been reported in *C. suis* worldwide (United States, several European countries, Israel, China) (Lenart et al., 2001; Dugan et al., 2004; Di Francesco et al., 2008; Borel et al., 2012; Schautteet et al., 2013; Joseph et al., 2016; Wanninger et al., 2016; Li et al., 2017; Seth-Smith et al., 2017). Therefore, tetracycline, a bacteriostatic protein synthesis inhibitor preventing the binding of bacterial aminoacyl-t-RNA to the mRNA-ribosome complex, is well suited as proof of concept for susceptibility assay development and evaluation, as there are clear, well-described features separating resistant from sensitive strains (Suchland et al., 2003; Donati et al., 2010). We further found that even sensitive strains are able to recover from tetracycline concentrations well above the MIC, which is indicative of heterotypic survival as reported by Suchland et al. (2003). Nonetheless, the observed infectivity pattern following recovery and continued exposure is markedly different between sensitive and resistant strains which is expected based on the MIC data. With application of the initial susceptibility phenotype analysis that can be performed within two to three workdays and subsequent MIC confirmation that can completed within two to three additional workdays, we showed that (a) simultaneous host cell seeding and chlamydial infection into culture medium with appropriate antibiotic dilutions yields similar results regarding susceptibility to more the time-consuming infection of confluent monolayers and that (b) cycloheximide treatment does not appear to influence MIC determination. With data from this study and those of other research groups (Suchland et al., 2003; Donati et al., 2016), we established a fast and simple screening method to detect tetracycline resistant strains. Our Tet screen allows the evaluation of ten strains per 24-well plate within 3 days with minimal use of consumables where cultures are treated with 0.5 μg/ml tetracycline and compared to mock-exposed cultures. If inclusion size/morphology and number are similar to that of the corresponding tetracycline-unexposed mock control, the strain is considered to be tetracycline resistant. If there are no, or only small, aberrant inclusions, the strain is considered to be tetracycline sensitive. All “intermediate” stages, such as few but regular sized inclusions, should be processed further by (a) determining the initial susceptibility phenotype, (b) performing subsequent MIC confirmation, and (c) performing the recovery assays. So far, no “intermediate” stages were detected, but a larger sample size must be evaluated to further validate this method.

Information regarding the susceptibility of *C. suis* to sulfonamides such as sulfamethoxazole, a bacteriostatic inhibitor of folate synthesis by competition with the substrate para-aminobenzoic acid (PABA) (Marwaha et al., 2014), is very limited. Reports generally state that *C. suis* appears to be sensitive to sulfonamides with the exception of a few tetracycline resistant strains (Andersen and Rogers, 1998; Sandoz and Rockey, 2010). These reports are contrary to our findings wherein all three field strains, of which two were sensitive to tetracycline, had sulfamethoxazole MICs of 64 μg/ml or higher. Moreover, MIC determination was only possible according to the MIC determination method by Suchland et al. (2003), because the inclusion number was not significantly influenced by sulfamethoxazole. Here, the recovery assay was crucial to confirm that the alteration in terms of size and morphology strongly impacted the infectivity upon recovery and continued exposure and therefore further served as a confirmation of the MIC. Interestingly, reporting results from a study on *C. trachomatis*, which defined the MIC as the concentration of sulfamethoxazole for which no inclusions are seen, the authors were able to determine susceptibilities for sulfamethoxazole in the range of 2–128 μg/ml (Hammerschlag, 1982). However, the authors specifically noted that only low inocula (≤1000 IFU) could be used to yield these results. Larger inocula did not yield an MIC according to their definition, but they showed a sulfamethoxazole concentration-dependent alteration in inclusion size and morphology. The susceptibility range (2–128 μg/ml) to sulfamethoxazole reported for *C. trachomatis* appears to be similar to that found for *C. suis* although the MIC of S45/6 was below 0.01 μg/ml. From the small number of strains investigated in our study, it appears that field strains are mostly resistant to sulfamethoxazole independent of resistance to tetracycline, while the *C. suis* type strain S45/6 is sulfamethoxazole sensitive. However, a larger sample size is necessary to confirm this finding. Moreover, these and other *C. suis* field strains should also be investigated for resistance to N-acylated sulfonamide derivatives because, despite sharing the structural core with sulfamethoxazole and sulfafurazole, they operate via a distinct working mechanism (Marwaha et al., 2014; Mojica et al., 2017): These antibiotics do not affect folate synthesis but instead bind directly to the 3-oxoacyl-[acyl carrier protein (ACP) synthase II (FabF)] thus inhibiting the essential type II fatty acid synthesis (FASII) pathway.

Given that almost every chlamydial species evaluated to date responds with the chlamydial stress response upon treatment with β-lactam antibiotics (Galasso and Manire, 1961; Tamura and Manire, 1968; Matsumoto and Manire, 1970; Beatty et al., 1994; Hogan et al., 2004; Wyrick, 2010; Schoborg, 2011) with few exceptions (Dumoux et al., 2013), it is not surprising that all four investigated *C. suis* strains develop persistence from PenG treatment. However, while this *in vitro* assay allowed us to detect and describe persistence in our cultures, more appropriate protocols are available to investigate the chlamydial stress response (Kintner et al., 2014; Leonard et al., 2015, 2016, 2017). Nevertheless, we showed that *C. suis* could recover from PenG exposure in a similar manner to *C. trachomatis*, though infectivity was reduced by more than 99% upon exposure to 1 U/ml in both chlamydial species (Kintner et al., 2014). Interestingly, while TEM analysis in the *C. trachomatis* study revealed large ABs that filled the inclusion after exposure with penicillin, we found few grossly enlarged ABs that did not fill the entire inclusion. Ultrastructural differences in the chlamydial stress response are primarily caused by different persistence inducers, host cells and the *Chlamydia* species (Hogan et al., 2004; Goellner et al., 2006; Mukhopadhyay et al., 2006; Schoborg, 2011). Therefore, host cells (HeLa vs. LLC-MK2) or the chlamydial species (*C. trachomatis* vs. *C. suis*) may have caused the ultrastructural differences in this study compared to Kintner et al. (2014). In contrast, HeLa cells infected with the porcine *C. pecorum* strain 1710S and PenG-exposed (Leonard et al., 2016, 2017) revealed similar inclusions to those present in PenG-exposed *C. suis* strains described in this study.

One of the limitations of our study is that we did not test our protocols using different host cells, unlike previous reports from other authors (Ehret and Judson, 1988; Suchland et al., 2003). Suchland et al. (2003) found that there are notable differences regarding the MIC for macrolides (azithromycin, erythromycin) for *Chlamydia* spp. depending on the cell line used, for example McCoy, HeLa, BGMK, HEp-2, HL, or Vero cells. But the same was not shown for tetracycline, ofloxacin, and doxycycline. The authors proposed that McCoy cells should be consistently used for *C. trachomatis* and HEp-2 for *C. pneumoniae* antibiotic susceptibility assays. So far, there is no specific recommendation for *C. suis* but McCoy, BGM, and LLC-MK2 cells have all been successfully used for isolation and antibiotic susceptibility assays (Dugan et al., 2004; Donati et al., 2010; Schautteet et al., 2013). For tetracyclines, cell-specific differences likely play a minor role as observed for *C. trachomatis* (Suchland et al., 2003), but no comparable study similar to that of Suchland et al. (2003) has been conducted for *C. suis* so far. Other potentially relevant factors not assessed in this study include medium constituents, incubation conditions and centrifugation protocols as well as timing/duration of antibiotic exposure and immunolabeling of inclusions (Giemsa, iodine, immunofluorescence) (Ehret and Judson, 1988). Additionally, the inoculum size is generally considered irrelevant for MIC determination but could influence the recovery assay if the inoculum size is below 5000 IFU/well (Suchland et al., 2003).

Furthermore, our study has primarily focused on the classic microscopic reading methods. In the future, it is vital consider alternative methods that were first published in the late 1990s such as the RT-PCR method (Cross et al., 1999). They found that smaller and/or aberrant inclusions still produce detectable levels of mRNA and are therefore potentially viable further supporting the results of the recovery assay applied in this study and the high MBC/MCC/MLC values from other protocols (Henning and Krauss, 1986; Webberley et al., 1987; Ehret and Judson, 1988; Japan Society of Chemotherapy, 1992a,b; Agacfidan et al., 1993; Suchland et al., 2003; Donati et al., 2010). Another method is the use of flow cytometry (Dessus-Babus et al., 1998), which was not considered to be as sensitive as the direct microscopic assessment method but still needs to be considered for its reproducibility and objective interpretation. Additionally, flow cytometry must be considered as a potential titration method (Käser et al., 2016) for the recovery assay as it has shown to be highly reproducible, faster with lower material cost than traditional titration methods.

## Conclusion

We propose new approaches to evaluate the antibiotic susceptibility of *C. suis* and other *Chlamydia* spp. by creating a consensus MIC based on inclusion number reduction and size/morphology alteration. This approach allows the determination of a susceptibility range for antibiotic agents and chlamydial species that have not been tested so far. Finally, we propose a simple and fast screening method to detect tetracycline resistant *C. suis* strains.

## Author Contributions

HM, CL, NB, and DD substantially contributed to the conception and design of the work, drafted and/or critically revised the manuscript, and finally approved the version to be published. HM, CL, and NB acquired, analyzed, and interpreted the data. All authors agreed to be accountable for all aspects of the work.

## Conflict of Interest Statement

The authors declare that the research was conducted in the absence of any commercial or financial relationships that could be construed as a potential conflict of interest.

## Footnotes

1 http://www.graphpad.com/quickcalcs/ttest1/ (accessed January 2018).

## References

[B1] Agacfidan A. Moncada J. Schachter J. (1993). In vitro activity of azithromycin (CP-62,993) against *Chlamydia trachomatis* and *Chlamydia pneumoniae*. *Antimicrob. Agents Chemother.* 37 1746–1748. 10.1128/AAC.37.9.1746 8239579 PMC188064

[B2] Andersen A. Rogers D. (1998). “Resistance to tetracycline and sulphadiazine in swine *C. trachomatis* isolates,” in *Proceedings for the 9th International Symposium on Human Chlamydial Infection*, Napa, CA, 313–316.

[B3] Beatty W. L. Morrison R. P. Byrne G. I. (1994). Persistent chlamydiae: from cell culture to a paradigm for chlamydial pathogenesis. *Microbiol. Rev.* 58 686–699. 7854252 10.1128/mr.58.4.686-699.1994 PMC372987

[B4] Borel N. Leonard C. Slade J. Schoborg R. V. (2016). Chlamydial antibiotic resistance and treatment failure in veterinary and human medicine. *Curr. Clin. Microbiol. Rep.* 3 10–18. 10.1007/s40588-016-0028-4 27218014 PMC4845085

[B5] Borel N. Pospischil A. Hudson A. P. Rupp J. Schoborg R. V. (2014). The role of viable but non-infectious developmental forms in chlamydial biology. *Front. Cell. Infect. Microbiol.* 4:97. 10.3389/fcimb.2014.00097 25105096 PMC4109588

[B6] Borel N. Regenscheit N. Di Francesco A. Donati M. Markov J. Masserey Y. (2012). Selection for tetracycline-resistant *Chlamydia suis* in treated pigs. *Vet. Microbiol.* 156 143–146. 10.1016/j.vetmic.2011.10.011 22036200

[B7] CLSI (2012). *Methods for Dilution Antimicrobial Susceptibility Tests for Bacteria that Grow Aerobicaly; Approved Standard, CLSI Document M07-A10*, 10th Edn. Wayne, PA: CLSI, 1–87. 10.4103/0976-237X.91790

[B8] Cross N. A. Kellock D. J. Kinghorn G. R. Taraktchoglou M. Bataki E. Oxley K. M. (1999). Antimicrobial susceptibility testing of *Chlamydia trachomatis* using a reverse transcriptase PCR-based method. *Antimicrob. Agents Chemother.* 43 2311–2313. 10471587 10.1128/aac.43.9.2311 PMC89469

[B9] De Puysseleyr K. De Puysseleyr L. Dhondt H. Geens T. Braeckman L. Morré S. A. (2014). Evaluation of the presence and zoonotic transmission of *Chlamydia suis* in a pig slaughterhouse. *BMC Infect. Dis.* 14:560. 10.1186/s12879-014-0560-x 25358497 PMC4216655

[B10] De Puysseleyr L. De Puysseleyr K. Braeckman L. Morré S. A. Cox E. Vanrompay D. (2015). Assessment of *Chlamydia suis* infection in pig farmers. *Transbound. Emerg. Dis.* 64 826–833. 10.1111/tbed.12446 26576707

[B11] Dean D. Rothschild J. Ruettger A. Kandel R. P. Sachse K. (2013). Zoonotic Chlamydiaceae species associated with trachoma, Nepal. *Emerg. Infect. Dis.* 19 1948–1955. 10.3201/eid1912.130656 24274654 PMC3840858

[B12] Deka S. Vanover J. Dessus-Babus S. Whittimore J. Howett M. K. Wyrick P. B. (2006). *Chlamydia trachomatis* enters a viable but non-cultivable (persistent) state within herpes simplex virus type 2 (HSV-2) co-infected host cells. *Cell. Microbiol.* 8 149–162. 10.1111/j.1462-5822.2005.00608.x 16367874

[B13] Dessus-Babus S. Belloc F. Bébéar C. M. Poutiers F. Lacombe F. Bébéar C. (1998). Antibiotic susceptibility testing for *Chlamydia trachomatis* using flow cytometry. *Cytometry* 31 37–44. 10.1002/(SICI)1097-0320(19980101)31:1<37::AID-CYTO5>3.0.CO;2-G 9450523

[B14] Di Francesco A. Donati M. Rossi M. Pignanelli S. Shurdhi A. Baldelli R. (2008). Tetracycline-resistant *Chlamydia suis* isolates in Italy. *Vet. Rec.* 163 251–252. 10.1136/vr.163.8.251 18723867

[B15] Donati M. Balboni A. Laroucau K. Aaziz R. Vorimore F. Borel N. (2016). Tetracycline susceptibility in *Chlamydia suis* pig isolates. *PLoS One* 11:e0149914. 10.1371/journal.pone.0149914 26913523 PMC4767523

[B16] Donati M. Di Francesco A. D’Antuono A. Delucca F. Shurdhi A. Moroni A. (2010). In vitro activities of several antimicrobial agents against recently isolated and genotyped *Chlamydia trachomatis* urogenital serovars D through K. *Antimicrob. Agents Chemother.* 54 5379–5380. 10.1128/AAC.00553-10 20855744 PMC2981272

[B17] Dugan J. Rockey D. D. Jones L. Andersen A. A. (2004). Tetracycline resistance in *Chlamydia suis* mediated by genomic islands inserted into the chlamydial inv-like gene. *Antimicrob. Agents Chemother.* 48 3989–3995. 10.1128/AAC.48.10.3989-3995.2004 15388463 PMC521927

[B18] Dumoux M. Le Gall S. M. Habbeddine M. Delarbre C. Hayward R. D. Kanellopoulos-Langevin C. (2013). Penicillin kills *Chlamydia* following the fusion of bacteria with lysosomes and prevents genital inflammatory lesions in *C. muridarum*-infected mice. *PLoS One* 8:e83511. 10.1371/journal.pone.0083511 24376710 PMC3871543

[B19] Ehret J. M. Judson F. N. (1988). Susceptibility testing of *Chlamydia trachomatis*: from eggs to monoclonal antibodies. *Antimicrob. Agents Chemother.* 32 1295–1299. 10.1128/AAC.32.9.1295 3058015 PMC175854

[B20] Galasso G. Manire G. (1961). Effect of antiserum and antibiotics on persistent infection of HeLa cells with meningopneumonitis virus. *J. Immunol.* 86 382–385. 13703027

[B21] Goellner S. Schubert E. Liebler-Tenorio E. Hotzel H. Saluz H. P. Sachse K. (2006). Transcriptional response patterns of *Chlamydophila psittaci* in different in vitro models of persistent infection. *Infect. Immun.* 74 4801–4808. 10.1128/IAI.01487-05 16861668 PMC1539575

[B22] Hammerschlag M. R. (1982). Activity of trimethoprim-sulfamethoxazole against *Chlamydia trachomatis* in vitro. *Rev. Infect. Dis.* 4 500–505. 10.1093/clinids/4.2.500 6981168

[B23] Henning K. Krauss H. (1986). Zur methodik der bestimmung der antibiotikumempfindlichkeit von chlamydien in vitro. *J. Vet. Med. Ser. B* 33 447–461. 10.1111/j.1439-0450.1986.tb00056.x 3788356

[B24] Hoffmann K. Schott F. Donati M. Di Francesco A. Hässig M. Wanninger S. (2015). Prevalence of chlamydial infections in fattening pigs and their influencing factors. *PLoS One* 10:e0143576. 10.1371/journal.pone.0143576 26619187 PMC4664257

[B25] Hogan R. J. Mathews S. A. Mukhopadhyay S. Summersgill J. T. Timms P. (2004). Chlamydial persistence: beyond the biphasic paradigm. *Infect. Immun.* 72 1843–1855. 10.1128/IAI.72.4.1843-1855.2004 15039303 PMC375192

[B26] Japan Society of Chemotherapy (1992a). Method for in vitro determination of chlamydial susceptibility (minimal lethal concentration; MLC) to antimicrobial agents. *Chemotherapy* 40 318–320.

[B27] Japan Society of Chemotherapy (1992b). Method for in vitro determination of chlamydial susceptibility (minimum inhibitory concentration; MIC) to antimicrobial agents. *Chemotherapy* 40 308–314.

[B28] Jeffrey B. M. Suchland R. J. Eriksen S. G. Sandoz K. M. Rockey D. D. (2013). Genomic and phenotypic characterization of in vitro-generated *Chlamydia trachomatis* recombinants. *BMC Microbiol.* 13:142. 10.1186/1471-2180-13-142 23786423 PMC3703283

[B29] Jorgensen J. H. Turnidge J. D. (2015). “Susceptibility test methods: dilution and disk diffusion methods,” in *Manual of Clinical Microbiology*, 11th Edn, eds Jorgensen J. H. Pfaller M. A. Carroll K. C. (Boston, MA: American Society of Microbiology), 1253–1273.

[B30] Joseph S. J. Marti H. Didelot X. Read T. D. Dean D. (2016). Tetracycline selective pressure and homologous recombination shape the evolution of *Chlamydia suis*: a recently identified zoonotic pathogen. *Genome Biol. Evol.* 8 2613–2623. 10.1093/gbe/evw182 27576537 PMC5010913

[B31] Kaltenboeck B. Kousoulas K. G. Storz J. (1993). Structures of and allelic diversity and relationships among the major outer membrane protein (ompA) genes of the four chlamydial species. *J. Bacteriol.* 175 487–502. 10.1128/jb.175.2.487-502.1993 8419295 PMC196164

[B32] Käser T. Pasternak J. A. Hamonic G. Rieder M. Lai K. Delgado-Ortega M. (2016). Flow cytometry as an improved method for the titration of *Chlamydiaceae* and other intracellular bacteria. *Cytom. Part A* 89 451–460. 10.1002/cyto.a.22822 26849001

[B33] Kintner J. Lajoie D. Hall J. Whittimore J. Schoborg R. V. (2014). Commonly prescribed Î2-lactam antibiotics induce *C. trachomatis* persistence/stress in culture at physiologically relevant concentrations. *Front. Cell. Infect. Microbiol.* 4:44 10.3389/fcimb.2014.00044 PMC3990100 24783061

[B34] Kutlin A. Roblin P. M. Hammerschlag M. R. (1999). In vitro activities of azithromycin and ofloxacin against *Chlamydia pneumoniae* in a continuous-infection model. *Antimicrob. Agents Chemother.* 43 2268–2272. 10471577 10.1128/aac.43.9.2268 PMC89459

[B35] Lenart J. Andersen A. A. Rockey D. D. (2001). Growth and development of tetracycline-resistant *Chlamydia suis*. *Antimicrob. Agents Chemother.* 45 2198–2203. 10.1128/AAC.45.8.2198-2203.2001 11451674 PMC90631

[B36] Leonard C. A. Dewez F. Borel N. (2016). Penicillin G-induced chlamydial stress response in a porcine strain of *Chlamydia pecorum*. *Int. J. Microbiol.* 2016:3832917. 10.1155/2016/3832917 26997956 PMC4779511

[B37] Leonard C. A. Schoborg R. V. Borel N. (2015). Damage/danger associated molecular patterns (DAMPs) modulate *Chlamydia pecorum* and *C. trachomatis* serovar E inclusion development in vitro. *PLoS One* 10:e0134943. 10.1371/journal.pone.0134943 26248286 PMC4527707

[B38] Leonard C. A. Schoborg R. V. Borel N. (2017). Productive and penicillin-stressed *Chlamydia pecorum* infection induces nuclear factor kappa B activation and interleukin-6 secretion in vitro. *Front. Cell. Infect. Microbiol.* 7:180. 10.3389/fcimb.2017.00180 28553623 PMC5425588

[B39] Lewis K. (2007). Persister cells, dormancy and infectious disease. *Nat. Rev. Microbiol.* 5 48–56. 10.1038/nrmicro1557 17143318

[B40] Li M. Jelocnik M. Yang F. Gong J. Kaltenboeck B. Polkinghorne A. (2017). Asymptomatic infections with highly polymorphic *Chlamydia suis* are ubiquitous in pigs. *BMC Vet. Res.* 13:370. 10.1186/s12917-017-1295-x 29191191 PMC5710075

[B41] Marti H. Kim H. Joseph S. J. Dojiri S. Read T. D. Dean D. (2017). Tet(C) gene transfer between *Chlamydia suis* strains occurs by homologous recombination after co-infection: implications for spread of tetracycline-resistance among Chlamydiaceae. *Front. Microbiol.* 8:156. 10.3389/fmicb.2017.00156 28223970 PMC5293829

[B42] Marwaha S. Uvell H. Salin O. Lindgren A. E. G. Silver J. Elofsson M. (2014). N-Acylated derivatives of sulfamethoxazole and sulfafurazole inhibit intracellular growth of *Chlamydia trachomatis*. *Antimicrob. Agents Chemother.* 58 2968–2971. 10.1128/AAC.02015-13 24566180 PMC3993265

[B43] Matsumoto A. Manire G. P. (1970). Electron microscopic observations on the effects of penicillin on the morphology of *Chlamydia psittaci*. *J. Bacteriol.* 101 278–285. 5413965 10.1128/jb.101.1.278-285.1970 PMC250478

[B44] Mojica S. A. Salin O. Bastidas R. J. Sunduru N. Hedenström M. Andersson C. D. (2017). N-acylated derivatives of sulfamethoxazole block *Chlamydia* fatty acid synthesis and interact with FabF. *Antimicrob. Agents Chemother.* 61:e00716-17. 10.1128/AAC.00716-17 28784680 PMC5610512

[B45] Mukhopadhyay S. Miller R. D. Sullivan E. D. Theodoropoulos C. Mathews S. A. Timms P. (2006). Protein expression profiles of *Chlamydia pneumoniae* in models of persistence versus those of heat shock stress response. *Infect. Immun.* 74 3853–3863. 10.1128/IAI.02104-05 16790757 PMC1489704

[B46] Phillips Campbell R. Kintner J. Whittimore J. Schoborg R. V. (2012). *Chlamydia muridarum* enters a viable but non-infectious state in amoxicillin-treated BALB/c mice. *Microbes Infect.* 14 1177–1185. 10.1016/j.micinf.2012.07.017 22943883 PMC3654801

[B47] Sandoz K. M. Rockey D. D. (2010). Antibiotic resistance in Chlamydiae. *Future Microbiol.* 5 1427–1442. 10.2217/fmb.10.96 20860486 PMC3075073

[B48] Schautteet K. de Clercq E. Miry C. van Groenweghe F. Delava P. Kalmar I. (2013). Tetracycline-resistant *Chlamydia suis* in cases of reproductive failure on Belgian, Cypriote and Israeli pig production farms. *J. Med. Microbiol.* 62 331–334. 10.1099/jmm.0.042861-0 23105027

[B49] Schautteet K. Vanrompay D. (2011). Chlamydiaceae infections in pig. *Vet. Res.* 42:29. 10.1186/1297-9716-42-29 21314912 PMC3041669

[B50] Schoborg R. V. (2011). Chlamydia persistence – a tool to dissect Chlamydia–host interactions. *Microbes Infect.* 13 649–662. 10.1016/j.micinf.2011.03.004 21458583 PMC3636554

[B51] Seth-Smith H. M. B. Wanninger S. Bachmann N. Marti H. Qi W. Donati M. (2017). The *Chlamydia suis* genome exhibits high levels of diversity, plasticity, and mobile antibiotic resistance: comparative genomics of a recent livestock cohort shows influence of treatment regimes. *Genome Biol. Evol.* 9 750–760. 10.1093/gbe/evx043 28338777 PMC5381551

[B52] Suchland R. J. Geisler W. M. Stamm W. E. (2003). Methodologies and cell lines used for antimicrobial susceptibility testing of *Chlamydia* spp. *Antimicrob. Agents Chemother.* 47 636–642. 10.1128/AAC.47.2.636-642.2003 12543671 PMC151736

[B53] Suchland R. J. Sandoz K. M. Jeffrey B. M. Stamm W. E. Rockey D. D. (2009). Horizontal transfer of tetracycline resistance among *Chlamydia* spp. in vitro. *Antimicrob. Agents Chemother.* 53 4604–4611. 10.1128/AAC.00477-09 19687238 PMC2772348

[B54] Tamura A. Manire G. P. (1968). Effect of penicillin on the multiplication of meningopneumonitis organisms (*Chlamydia psittaci*). *J. Bacteriol.* 96 875–880. 5686015 10.1128/jb.96.4.875-880.1968 PMC252392

[B55] Wanninger S. Donati M. Di Francesco A. Hässig M. Hoffmann K. Seth-Smith H. M. B. (2016). Selective pressure promotes tetracycline resistance of *Chlamydia suis* in fattening pigs. *PLoS One* 11:e0166917. 10.1371/journal.pone.0166917 27893834 PMC5125646

[B56] Webberley J. M. Matthews R. S. Andrews J. M. Wise R. (1987). Commercially available fluorescein-conjugated monoclonal antibody for determining the in vitro activity of antimicrobial agents against *Chlamydia trachomatis*. *Eur. J. Clin. Microbiol.* 6 587–589. 10.1007/BF02014256 2449348

[B57] Wyrick P. B. (2010). *Chlamydia trachomatis* persistence in vitro: an overview. *J. Infect. Dis.* 201(Suppl.), S88–S95. 10.1086/652394 20470046 PMC2878585

[B58] Xue Y. Zheng H. Mai Z. Qin X. Chen W. Huang T. (2017). An in vitro model of azithromycin-induced persistent *Chlamydia trachomatis* infection. *FEMS Microbiol. Lett.* 364:fnx145. 10.1093/femsle/fnx145 28854672

[B59] Zheng H. Xue Y. Bai S. Qin X. Lu P. Yang B. (2015). Association of the in vitro susceptibility of clinical isolates of *Chlamydia trachomatis* with serovar and duration of antibiotic exposure. *Sex. Transm. Dis.* 42 115–119. 10.1097/OLQ.0000000000000241 25668641

