# Supplementary material for: Evaluating the Antibiotic Susceptibility of Chlamydia – New Approaches for in Vitro Assays
Source: Front Microbiol. 2018 Jul 3;9:1414. doi: 10.3389/fmicb.2018.01414 (PMC6037721; doi:10.3389/fmicb.2018.01414)
Supplement: Supplementary file 1 [file Data_Sheet_1.pdf]

Evaluating the antibiotic susceptibility of *Chlamydia* – new approaches for *in vitro* assays

Hanna Marti[1, 2], Nicole Borel[1], Deborah Dean[2, 3, 4], Cory Ann Leonard[1, *, #]

[1] Institute for Veterinary Pathology, Vetsuisse Faculty, University of Zurich, Zurich, Switzerland
[2] Center for Immunobiology and Vaccine Development, UCSF Benioff Children's Hospital Oakland Research Institute, Oakland, California, USA
[3] Joint Graduate Program in Bioengineering, University of California, San Francisco, California, USA, and University of California, Berkeley, Berkeley, California, USA
[4] School of Medicine, University of California, San Francisco, California, USA

Text S1. Complete *in vitro* antibiotic susceptibility assay protocol.

Shown is the detailed protocol for the complete *in vitro* antibiotic susceptibility assay for tetracycline, sulfamethoxazole and penicillin. Example stock concentrations, dilution schemes and plate layouts, exactly as used to generate the data presented, are included for illustration. In addition, suggestions regarding assay optimization are included.

Table of content

1 General information 2
1.1 Important definitions and parameters (all antibiotics) 2
1.2 Material / Instruments (all experiments) 2
1.3 Immunofluorescence assay (IFA) labeling (200 µl per well for each solution) 3
1.4 Immunofluorescence analysis 3
2 Tetracycline (Tet) 5
2.1 Tetracycline resistance screen (Tet Screen) 5
2.2 Initial susceptibility phenotype assay and Recovery assay (Part I) 6
2.3 Recovery assay (Part II) 9
2.4 MIC confirmation 10
3 Sulfamethoxazole (SULF)* 11
3.1 Initial susceptibility phenotype / Recovery assay (Part I) 11
3.2 Recovery assay (Part II) 13
3.3 MIC confirmation 14
4 Penicillin G (PenG) 15
4.1 Initial susceptibility phenotype / Recovery assay (Part I) 15
4.2 Recovery assay (Part II) 16
4.3 MIC confirmation / Inclusion size analysis 16

1 General information

1.1 Important definitions and parameters (all antibiotics)

Minimal inhibitory concentration (MIC) is defined as the consensus of the MIC as defined by Donati et al. (2010. AAC 54(12): 5379-5380. doi:10.1128/AAC.00553-10) and as defined by Suchland et al. (2003. AAC 47(2): 636-42. doi: 10.1128/AAC.47.2.636-642.2003), unless one parameter could not be employed.

- MIC (Donati) = "The lowest antibiotic concentration that **reduced the number of inclusions more than 90%** compared with the level for antibiotic-free controls." (p. 5380)
- MIC (Suchland) = "The transition point MIC (MIC_{TP}) was defined as the concentration of antibiotic in which **90% or more of the inclusions were altered in size and morphology**. The MIC was defined as the concentration of antibiotic that is twofold more concentrated than the MIC_{TP}." (p. 637)

The recovery assay compares chlamydial recovery upon discontinuation of antibiotic exposure for 48 h following exposure to the antibiotic for 48 h (rec) to both continuous antibiotic exposure for 96 h (exp) and to the mock-exposed control (mock). Infectivity is expressed as IFU/ml (semi-quantitative analysis) and is expressed through the following three parameters.

1) The *resistance potential* represents the degree to which *Chlamydia* resist continuous exposure to the antibiotic in question (exp to mock).
   It indicates the highest antibiotic concentration at which exp cultures exhibit infectivity equivalent to a) >25% or b) >10% of mock-exposed culture infectivity.
2) The *recovery potential* represents the degree to which *Chlamydia* recover from exposure (rec to mock).
   It indicates the highest antibiotic concentration at which rec cultures exhibit infectivity equivalent to a) >1% or b) >10% of mock-exposed culture infectivity.
3) *Survival after continued exposure* directly compares the infectivity of continuously exposed to recovered cultures (exp to rec).
   It provides the highest antibiotic concentration at which exp cultures exhibit infectivity equivalent to a) >1% or b) >10% of rec culture infectivity.

1.2 Material / Instruments (all experiments)

- LLC-MK2 cells (IZSLER Brescia, Italy; kindly provided by Donati M)
  - Cell growth media: 500 ml Eagle's minimum essential medium (MEM, Gibco, Thermo Fisher Scientific cat# 12360-038, Invitrogen, Carlsbad, CA, USA), 50 ml heat-inactivated fetal calf serum (FCS, BioConcept, Allschwil, Switzerland), 5 ml L-glutamine (GlutaMAX, 200 mM, Gibco, Thermo Fisher Scientific) and 6 ml glucose (stock: 0.06 g/ml) (Sigma-Aldrich Co., St. Louis, MO, USA) [Final concentrations: 8.9% FCS, 2.15 mM L-glutamine, 1.5 mg/ml glucose (non-supplemented MEM has 1 mg/ml glucose)]
  - Chlamydiae cultivation media: 500 ml MEM, 100 ml FCS, 5 ml L-glutamine, 2 g glucose with or without 0.7 ml cycloheximide (stock: 1 mg/ml; Sigma-Aldrich)* [Final concentrations: 16.5% FCS, 1.7 mM L-glutamine, 9.9 mg/ml glucose (non-supplemented MEM has 1 mg/ml glucose)]

    **NOTE: For freshly isolated chlamydial stocks the media can be supplemented with 4 ml fungizone (stock: 250 μg/ml, Gibco), 0.5 ml gentamycin (stock: 50 mg/ml, Gibco) and 5 ml vancomycin (stock: 10 mg/ml, Gibco) to prevent contaminating microbial overgrowth*

- Antibiotics
    - Tetracycline (hydrochloride, powder; Sigma-Aldrich, T7660-25G)
      ⇒ stocks prepared in deionized H_2O (final concentration: 10 mg/ml), frozen at -20°C
    - Sulfamethoxazole (Santa Cruz Biotechnology, LabForce, #sc-208405)
      ⇒ stocks prepared in DMSO (final concentration: 50 mg/ml), frozen at -20°C
    - Penicillin G (sodium salt; Sigma-Aldrich, P3032-25MU; potency ≥ 1477 U/mg)
      ⇒ stocks prepared in deionized H_2O (final concentration: 20'000 units (U)/ml; e.g. 135.4 mg in 10 ml), frozen at -20°C
- *For recovery assay / MIC confirmation / Tet Screen:* 24-well plates and glass coverslips
- *For initial susceptibility phenotype, recovery assay:* 96-well plates
- *Other consumables*: methanol, Pasteur pipettes, serological pipettes, standard pipettes and tips
- Centrifuge (e.g. Sorvall LegendXTR)

- **Immunofluorescence assay (IFA) antibodies**
    - *Chlamydiaceae* family-specific mouse monoclonal antibody against the chlamydial lipopolysaccharide (LPS, Clone ACI-P; Progen, Heidelberg, Germany; 1:200)
    - Alexa Fluor 488-conjugated secondary goat anti-mouse antibody (Molecular Probes, Eugene, OR, USA; 1:500)
    - Labeling of host and chlamydial DNA: 1 μg/mL 4′,6-diamidino-2′-phenylindole dihydrochloride (DAPI, Molecular Probes)

1.3 Immunofluorescence assay (IFA) labeling (200 μl per well for each solution)

1. 1% bovine serum albumin (BSA, heat shock fraction, pH 5.2, ≥96%, Sigma-Aldrich, cat# A3912-5009) in Phosphate-buffered saline (PBS; Gibco, no calcium, no magnesium) as blocking solution: 30 min at room temperature
2. 1:200 primary *Chlamydia*-LPS antibody in blocking solution: 60 min at room temperature (RT)
3. Wash 2-3x with PBS
4. 1 µg/ml DAPI & 1:500 secondary Alexa goat anti-mouse antibody: 45 min at RT
5. Wash 2-3x with PBS
6. Mounting of coverslips (e.g. with FluoreGuard mounting medium; Hard Set; ScyTek Laboratories Inc., Logan, UT, USA) onto slides for analysis

- *For recovery assay / MIC confirmation / Tet Screen:* Fluorescence microscope to evaluate coverslips on glass slides (e.g. Leica DMLB fluorescence microscope)
- *For Initial susceptibility phenotype:* Fluorescence microscope to evaluate 96-well plates (e.g. Nikon Eclipse T*i*)

1.4 Immunofluorescence analysis

- *Inclusion number (MIC confirmation - MIC Donati)*: The mean number of inclusions per field is determined for 30 fields at 200x magnification and the mean is compared in antibiotic-exposed cultures versus the mock-exposed cultures. The lowest treatment concentration in which the mean number of inclusions is reduced by more than 90% of the mock mean value is considered to be the MIC.
  For the initial susceptibility phenotype, no semiquantitative evaluation of the mean number of inclusions per field was determined. Instead, approximate inclusion number was determined at 200x and 400x and generalized inclusion number reduction was used to determine the MIC.

- *Inclusion size and morphology (MIC confirmation – MIC Suchland):* The MIC according to Suchland et al. (2003) is based on two different criteria: inclusion size and morphology, neither of which can be as easily quantified as the inclusion number due to possible variability. Despite this drawback, MIC determination is possible as the change from normal to altered inclusions in 90% of the inclusions is abrupt rather than gradual. In order to quantify this change, we semiquantitatively determine the mean inclusion size. For that, 50 randomly selected inclusions are evaluated in at least 10 fields (400x magnification) per condition and the area (in μm^2) is calculated using BonTec measuring and archiving software (BonTec, Bonn, Germany). Representative microscopic images are captured using BonTec software (BonTec) and a UI-2250SE-C-HQ camera (uEye, IDS Imaging Development Systems GmbH, Obersulm, Germany) as described previously (Leonard et al. 2015. PLoS One 10(8):e0134943. doi: 10.1371/journal.pone.0134943). For conditions with only few or very small inclusions, up to 20 inclusions are analyzed for size and morphology if possible. In parallel, we qualitatively evaluate inclusion morphology. The following criteria are used to classify the inclusion morphology as altered compared to control inclusion morphology: size and/or the presence of aberrant bodies (ABs, aberrant inclusion bodies; diameter ≥ 2 µm, [Matsumoto and Manire. 1970. J Bacteriolo 101(1):278-285]) as shown in the figure below (this study, strain SWA-14, scale bar = 5 µm). Micro-inclusions are defined as inclusions with an area of less than 15 μm^2.

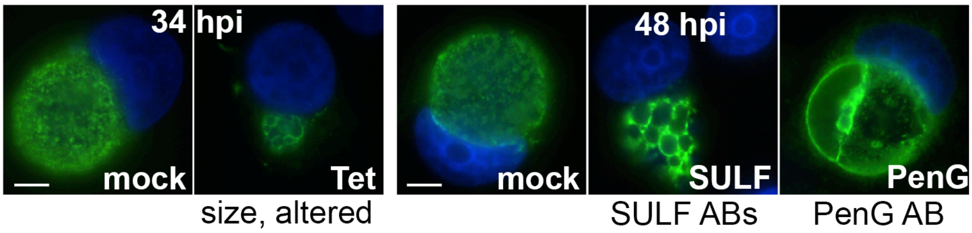

Taken together, MIC (Suchland) is considered to be the concentration 2-fold above the lowest treatment concentration in which inclusion size and/or morphology are altered, compared to the mock, in over 90% of inclusions.

For the initial susceptibility phenotype, no semiquantitative evaluation of the average inclusion size was performed. Instead, inclusion size and morphology was determined at 200x and 400x and generalized inclusion size/morphology reduction or alteration was used to determine the MIC.

- *Inclusion forming units per ml (IFU/ml) for the recovery assay:* For this study, the mean number of inclusions per field for 30 fields was determined at 200x magnification. The IFU/ml was calulated by multiplying the mean inculsions per field by the number of optic fields per well and dividing the resulting total inclusions per well by the volume, in ml, of chlamydial inoculum added to the well. This gives the IFU/ml in the scraped sample. If only a few inclusions were visible per coverslip, all inclusions were counted per coverslip and the formula was adjusted by an additional multiplication factor that corrected for the size difference of the coverslip compared to the well. Alternatively, other methods to determine the infectivity such as real-time PCR (De Puysseleyr et al. 2014. PloS One 9(5): e96704. doi: 10.1371/journal.pone.0096704; Wiggins et al. 2009. Clin. Microbiol 47(6): 1824-1829. doi:10.1128/JCM.00005-09), can be employed.

2 Tetracycline (Tet)

2.1 Tetracycline resistance screen (Tet Screen)

Up to 10 strains can be tested in two 24-well plates if both 0.125 and 0.5 µg/ml of tetracycline are evaluated.
For each plate and tetracycline concentration, a tetracycline resistant (e.g. R19 (Dugan et al. 2004. AAC 48(10): 3989-95. doi: 10.1128/AAC.48.10.3989-3995.2004), SWA-141) and a tetracycline sensitive (e.g. S45/6, SWA-14, 86) control must be included.

*Protocol**
Important note concerning medium: Chlamydia cultivation medium **contains cycloheximide.*

1. *1-3 days prior to start* - Prepare LLC-MK2 monolayers:
   Seed 2x 24-well plates for a confluent monolayer on glass coverslips

2. Day 1 (start infection)
   Prepare sufficient chlamydial suspension (1 ml per well, multiplicity of infection [MOI] approximately 0.5) for each strain including one tetracycline sensitive and one tetracycline resistant control strain.
   a) Add 1 ml of chlamydial suspension of each strain to four wells; 2 wells per 24-well plate (numerical values shown indicate tetracycline concentrations in the media that will be added immediately after centrifugation, mock = media only):

   Tet Screen, Plate 1 (S = sensitive control; R = resistant control; lowercase letters represent individual strains evaluated)

a Mock	a 0.125	e Mock	e 0.125	i Mock	i 0.125
b Mock	b 0.125	f Mock	f 0.125	j Mock	j 0.125
c Mock	c 0.125	g Mock	g 0.125	S Mock	S 0.125
d Mock	d 0.125	h Mock	h 0.125	R Mock	R 0.125

   Tet Screen, Plate 2 (grey = sensitive control; black = resistant control)

a Mock	a 0.5	e Mock	e 0.5	i Mock	i 0.5
b Mock	b 0.5	f Mock	f 0.5	j Mock	j 0.5
c Mock	c 0.5	g Mock	g 0.5	S Mock	S 0.5
d Mock	d 0.5	h Mock	h 0.5	R Mock	R 0.5

   b) Centrifuge plates for 1 h, 1000 g, 25 °C
   c) *During centrifugation*: prepare appropriate tetracycline concentrations in sterile Eppendorf or Falcon tubes by serial dilution from 10 mg/ml stock solution*:

**Tetracycline (µg/ml)**	**256**	**32**	**4**	**0.5**	**0.125**
Tetracycline (µl) from stock / previous tube	38.4 (from stock)	1000 **(1:8)**	1000 **(1:8)**	2000 **(1:8)**	3000 **(1:4)**
Cultivation medium (µl)	1461.6	7000	7000	14000	9000

   **NOTE: Protect tetracycline from light; Stock solutions are stable for up to one year at -20°C storage but should be retested every 3-6 months; tetracycline dilutions in media are stable for up to 30 days if stored at -20°C; freeze-thaw cycles do not diminish tetracycline stability for up to three cycles (data not shown).*

   d) After centrifugation, remove chlamydial suspension and add 1 ml per well of medium with appropriate tetracycline concentration, or 1 ml of medium alone for mock-exposed control
   e) Incubate plates for 34 h at 37 °C (5% CO_2)

3. Day 2 (fixation of plates for Tet screen)
    a) Remove culture medium and fix plates in methanol (cooled to -20 °C, 10 min)
    b) Replace methanol with PBS and store at 4 °C until IFA labeling is carried out

Tet Screen analysis

Check for the presence or absence of inclusions in the tetracycline-exposed cultures:

1. Resistant strains: inclusion size/morphology and inclusion number = mock
2. Sensitive strains: no inclusions, or only micro-inclusions, in 0.5 µg/ml tetracycline
3. "Intermediate" strains: reduced number of regular sized inclusions or a normal number of morphologically altered inclusions compared to the mock.

Unless exact MIC determination is required, sensitive and resistant strains do not need to be processed further (initial susceptibility phenotype, MIC confirmation, recovery assay). "Intermediate" strains should always be further processed with the *in vitro* antibiotic susceptibility assay (initial susceptibility phenotype assay, MIC confirmation, recovery assay).

2.2 *Initial susceptibility phenotype assay and Recovery assay (Part I)*

Up to 4 strains can be evaluated in three 96-well plates. For the initial susceptibility phenotype assay, a tetracycline resistant (e.g. R19 (Dugan et al. 2004. AAC 48(10): 3989-95. doi: 10.1128/AAC.48.10.3989-3995.2004), SWA-141) and a tetracycline sensitive control (e.g. S45/6, SWA-14, SWA-86) should be included.

*Protocol**

Important note concerning medium: Chlamydia cultivation medium* ***does not contain* *cycloheximide.*

1. *Preparation of tetracycline dilutions (2-fold serial dilutions, multichannel pipette) in the 96 well plates that will later be used for the initial susceptibility phenotype assay and the recovery assay (Part I; Days 1 and 3)*
    a) Prepare at least 5 ml of tetracycline with a final concentration of 16 µg/ml (8 µl of 10 mg/ml tetracycline stock + 4'792 µl cultivation medium)
    b) Prepare tetracycline dilutions in 96-well plates as shown below Dilution only if applicable, discarding 100 µl from well 12

Initial susceptibility phenotype Plate:

Well	1	2	3	4	5	6	7	8	9	10	11	12
**Conc. (µg/ml)**	Mock	16	8	4	2	1	0.5	0.25	0.13	0.06	0.03	1.5E-2
**Tetracycline**	None	200 µl	100 µl	100 µl	100 µl	100 µl	100 µl	100 µl	100 µl	100 µl	100 µl	100 µl
**Medium**	100 µl	0 µl	100 µl	100 µl	100 µl	100 µl	100 µl	100 µl	100 µl	100 µl	100 µl	100 µl
**Conc. (µg/ml)**	Mock	16	8	4	2	1	0.5	0.25	0.13	0.06	0.03	1.5E-2
**Tetracycline**	None	200 µl	100 µl	100 µl	100 µl	100 µl	100 µl	100 µl	100 µl	100 µl	100 µl	100 µl
**Medium**	100 µl	0 µl	100 µl	100 µl	100 µl	100 µl	100 µl	100 µl	100 µl	100 µl	100 µl	100 µl
**Conc. (µg/ml)**	Mock	16	8	4	2	1	0.5	0.25	0.13	0.06	0.03	1.5E-2
**Tetracycline**	None	200 µl	100 µl	100 µl	100 µl	100 µl	100 µl	100 µl	100 µl	100 µl	100 µl	100 µl
**Medium**	100 µl	0 µl	100 µl	100 µl	100 µl	100 µl	100 µl	100 µl	100 µl	100 µl	100 µl	100 µl
**Conc. (µg/ml)**	Mock	16	8	4	2	1	0.5	0.25	0.13	0.06	0.03	1.5E-2
**Tetracycline**	None	200 µl	100 µl	100 µl	100 µl	100 µl	100 µl	100 µl	100 µl	100 µl	100 µl	100 µl
**Medium**	100 µl	0 µl	100 µl	100 µl	100 µl	100 µl	100 µl	100 µl	100 µl	100 µl	100 µl	100 µl
**Conc. (µg/ml)**	Mock	16	8	4	2	1	0.5	0.25	0.13	0.06	0.03	1.5E-2
**Tetracycline**	None	200 µl	100 µl	100 µl	100 µl	100 µl	100 µl	100 µl	100 µl	100 µl	100 µl	100 µl
**Medium**	100 µl	0 µl	100 µl	100 µl	100 µl	100 µl	100 µl	100 µl	100 µl	100 µl	100 µl	100 µl
**Conc. (µg/ml)**	Mock	16	8	4	2	1	0.5	0.25	0.13	0.06	0.03	1.5E-2
**Tetracycline**	None	200 µl	100 µl	100 µl	100 µl	100 µl	100 µl	100 µl	100 µl	100 µl	100 µl	100 µl
**Medium**	100 µl	0 µl	100 µl	100 µl	100 µl	100 µl	100 µl	100 µl	100 µl	100 µl	100 µl	100 µl
**Conc. (µg/ml)**	Mock	16	8	4	2	1	0.5	0.25	0.13	0.06	0.03	1.5E-2
**Tetracycline**	None	200 µl	100 µl	100 µl	100 µl	100 µl	100 µl	100 µl	100 µl	100 µl	100 µl	100 µl
**Medium**	100 µl	0 µl	100 µl	100 µl	100 µl	100 µl	100 µl	100 µl	100 µl	100 µl	100 µl	100 µl
**Conc. (µg/ml)**	Mock	16	8	4	2	1	0.5	0.25	0.13	0.06	0.03	1.5E-2
**Tetracycline**	None	200 µl	100 µl	100 µl	100 µl	100 µl	100 µl	100 µl	100 µl	100 µl	100 µl	100 µl
**Medium**	100 µl	0 µl	100 µl	100 µl	100 µl	100 µl	100 µl	100 µl	100 µl	100 µl	100 µl	100 µl

Recovery assay (Day 1) Plate:

Well	1	2	3	4	5	6	7	8	9	10	11	12
**Conc. (µg/ml)**	Mock	16	8	4	2	1	0.5	0.25	0.13	0.06	0.03	1.5E-2
**Tetracycline**	None	200 µl	100 µl	100 µl	100 µl	100 µl	100 µl	100 µl	100 µl	100 µl	100 µl	100 µl
**Medium**	100 µl	0 µl	100 µl	100 µl	100 µl	100 µl	100 µl	100 µl	100 µl	100 µl	100 µl	100 µl
**Conc. (µg/ml)**	Mock	16	8	4	2	1	0.5	0.25	0.13	0.06	0.03	1.5E-2
**Tetracycline**	None	200 µl	100 µl	100 µl	100 µl	100 µl	100 µl	100 µl	100 µl	100 µl	100 µl	100 µl
**Medium**	100 µl	0 µl	100 µl	100 µl	100 µl	100 µl	100 µl	100 µl	100 µl	100 µl	100 µl	100 µl
**Conc. (µg/ml)**	Mock	16	8	4	2	1	0.5	0.25	0.13	0.06	0.03	1.5E-2
**Tetracycline**	None	200 µl	100 µl	100 µl	100 µl	100 µl	100 µl	100 µl	100 µl	100 µl	100 µl	100 µl
**Medium**	100 µl	0 µl	100 µl	100 µl	100 µl	100 µl	100 µl	100 µl	100 µl	100 µl	100 µl	100 µl
**Conc. (µg/ml)**	Mock	16	8	4	2	1	0.5	0.25	0.13	0.06	0.03	1.5E-2
**Tetracycline**	None	200 µl	100 µl	100 µl	100 µl	100 µl	100 µl	100 µl	100 µl	100 µl	100 µl	100 µl
**Medium**	100 µl	0 µl	100 µl	100 µl	100 µl	100 µl	100 µl	100 µl	100 µl	100 µl	100 µl	100 µl
**Conc. (µg/ml)**	Mock	16	8	4	2	1	0.5	0.25	0.13	0.06	0.03	1.5E-2
**Tetracycline**	None	200 µl	100 µl	100 µl	100 µl	100 µl	100 µl	100 µl	100 µl	100 µl	100 µl	100 µl
**Medium**	100 µl	0 µl	100 µl	100 µl	100 µl	100 µl	100 µl	100 µl	100 µl	100 µl	100 µl	100 µl
**Conc. (µg/ml)**	Mock	16	8	4	2	1	0.5	0.25	0.13	0.06	0.03	1.5E-2
**Tetracycline**	None	200 µl	100 µl	100 µl	100 µl	100 µl	100 µl	100 µl	100 µl	100 µl	100 µl	100 µl
**Medium**	100 µl	0 µl	100 µl	100 µl	100 µl	100 µl	100 µl	100 µl	100 µl	100 µl	100 µl	100 µl
**Conc. (µg/ml)**	Mock	16	8	4	2	1	0.5	0.25	0.13	0.06	0.03	1.5E-2
**Tetracycline**	None	200 µl	100 µl	100 µl	100 µl	100 µl	100 µl	100 µl	100 µl	100 µl	100 µl	100 µl
**Medium**	100 µl	0 µl	100 µl	100 µl	100 µl	100 µl	100 µl	100 µl	100 µl	100 µl	100 µl	100 µl
**Conc. (µg/ml)**	Mock	16	8	4	2	1	0.5	0.25	0.13	0.06	0.03	1.5E-2
**Tetracycline**	None	200 µl	100 µl	100 µl	100 µl	100 µl	100 µl	100 µl	100 µl	100 µl	100 µl	100 µl
**Medium**	100 µl	0 µl	100 µl	100 µl	100 µl	100 µl	100 µl	100 µl	100 µl	100 µl	100 µl	100 µl

Recovery assay (Day 3) Plate:

Well	1	2	3	4	5	6	7	8	9	10	11	12
**Recovery**	Mock	No Tet	No Tet	No Tet	No Tet	No Tet	No Tet	No Tet	No Tet	No Tet	No Tet	No Tet
**Tetracycline**	None	None	None	None	None	None	None	None	None	None	None	None
**Medium**	150 µl	150 µl	150 µl	150 µl	150 µl	150 µl	150 µl	150 µl	150 µl	150 µl	150 µl	150 µl
**Cont. Exp.**	Mock	8	4	2	1	0.5	0.25	0.13	0.06	0.03	1.5E-2	7.8E-3
**Tetracycline**	None	150 µl	150 µl	150 µl	150 µl	150 µl	150 µl	150 µl	150 µl	150 µl	150 µl	150 µl
**Medium**	150 µl	150 µl	150 µl	150 µl	150 µl	150 µl	150 µl	150 µl	150 µl	150 µl	150 µl	150 µl
**Recovery**	Mock	No Tet	No Tet	No Tet	No Tet	No Tet	No Tet	No Tet	No Tet	No Tet	No Tet	No Tet
**Tetracycline**	None	None	None	None	None	None	None	None	None	None	None	None
**Medium**	150 µl	150 µl	150 µl	150 µl	150 µl	150 µl	150 µl	150 µl	150 µl	150 µl	150 µl	150 µl
**Cont. Exp.**	Mock	8	4	2	1	0.5	0.25	0.13	0.06	0.03	1.5E-2	7.8E-3
**Tetracycline**	None	150 µl	150 µl	150 µl	150 µl	150 µl	150 µl	150 µl	150 µl	150 µl	150 µl	150 µl
**Medium**	150 µl	150 µl	150 µl	150 µl	150 µl	150 µl	150 µl	150 µl	150 µl	150 µl	150 µl	150 µl
**Recovery**	Mock	No Tet	No Tet	No Tet	No Tet	No Tet	No Tet	No Tet	No Tet	No Tet	No Tet	No Tet
**Tetracycline**	None	None	None	None	None	None	None	None	None	None	None	None
**Medium**	150 µl	150 µl	150 µl	150 µl	150 µl	150 µl	150 µl	150 µl	150 µl	150 µl	150 µl	150 µl
**Cont. Exp.**	Mock	8	4	2	1	0.5	0.25	0.13	0.06	0.03	1.5E-2	7.8E-3
**Tetracycline**	None	150 µl	150 µl	150 µl	150 µl	150 µl	150 µl	150 µl	150 µl	150 µl	150 µl	150 µl
**Medium**	150 µl	150 µl	150 µl	150 µl	150 µl	150 µl	150 µl	150 µl	150 µl	150 µl	150 µl	150 µl
**Recovery**	Mock	No Tet	No Tet	No Tet	No Tet	No Tet	No Tet	No Tet	No Tet	No Tet	No Tet	No Tet
**Tetracycline**	None	None	None	None	None	None	None	None	None	None	None	None
**Medium**	150 µl	150 µl	150 µl	150 µl	150 µl	150 µl	150 µl	150 µl	150 µl	150 µl	150 µl	150 µl
**Cont. Exp.**	Mock	8	4	2	1	0.5	0.25	0.13	0.06	0.03	1.5E-2	7.8E-3
**Tetracycline**	None	150 µl	150 µl	150 µl	150 µl	150 µl	150 µl	150 µl	150 µl	150 µl	150 µl	150 µl
**Medium**	150 µl	150 µl	150 µl	150 µl	150 µl	150 µl	150 µl	150 µl	150 µl	150 µl	150 µl	150 µl

**NOTE: The minimum number of plates that must be prepared for one experiment is three. Shaded wells indicate the minimum number of wells required for evaluation of a single strain. Dilutions can be stored at -20°C for up to 30 days with similar results to freshly prepared plates (data not shown).*

c) Freeze plates until use (no longer than 30 days)

2. Day 1 (seed & infection)
   a) Split cells into cycloheximide-free medium and count cells to prepare a cell suspension with **300,000 cells/ml** (⇒ 30,000 cells per well)
   b) Add chlamydial stock for a final MOI of 0.5
   c) Add 100 µl of Chlamydia/cell mix per well to 100 µl prepared antibiotics (see below)
   d) Centrifuge plates for 1 h, 1000 g, 25 °C
   e) Incubate plates for 34 h at 37 °C (5% CO_2)

Initial susceptibility phenotype plate (final tetracycline concentration in µg/ml)

Mock	8	4	2	1	0.5	0.25	0.125	0.06	0.03	0.015	7.8E-3
Mock	8	4	2	1	0.5	0.25	0.125	0.06	0.03	0.015	7.8E-3
Mock	8	4	2	1	0.5	0.25	0.125	0.06	0.03	0.015	7.8E-3
Mock	8	4	2	1	0.5	0.25	0.125	0.06	0.03	0.015	7.8E-3
Mock	8	4	2	1	0.5	0.25	0.125	0.06	0.03	0.015	7.8E-3
Mock	8	4	2	1	0.5	0.25	0.125	0.06	0.03	0.015	7.8E-3
Mock	8	4	2	1	0.5	0.25	0.125	0.06	0.03	0.015	7.8E-3
Mock	8	4	2	1	0.5	0.25	0.125	0.06	0.03	0.015	7.8E-3

Grey: sensitive control; blue to purple: strains 1-4; black: resistant control

Recovery assay (final concentration in µg/ml)

Mock	8	4	2	1	0.5	0.25	0.125	0.06	0.03	0.015	7.8E-3
Mock	8	4	2	1	0.5	0.25	0.125	0.06	0.03	0.015	7.8E-3
Mock	8	4	2	1	0.5	0.25	0.125	0.06	0.03	0.015	7.8E-3
Mock	8	4	2	1	0.5	0.25	0.125	0.06	0.03	0.015	7.8E-3
Mock	8	4	2	1	0.5	0.25	0.125	0.06	0.03	0.015	7.8E-3
Mock	8	4	2	1	0.5	0.25	0.125	0.06	0.03	0.015	7.8E-3
Mock	8	4	2	1	0.5	0.25	0.125	0.06	0.03	0.015	7.8E-3
Mock	8	4	2	1	0.5	0.25	0.125	0.06	0.03	0.015	7.8E-3

Blue to purple: strains 1-4 in duplicate

3. Day 2 (fixation plate for initial susceptibility phenotype); at 34 hours post infection (hpi)
   - Remove culture medium, add chilled (-20 °C) methanol for 10 min, replace methanol with sterile PBS and store at 4 °C until IFA labeling is carried out

4. Day 3 (wash plates for recovery assay & analysis of initial susceptibility phenotype); at 48 hpi
   a) Thaw tetracycline dilutions of the "Recovery assay – Day 3 plate" in incubator (37 °C)
   b) Wash the recovery plate ("Day 1") 2x with *Chlamydia* cultivation medium
   c) Transfer the media from the "Recovery assay – Day 3 plate" to the recovery plate
   d) Incubate plates for 34 h at 37 °C (5% CO_2)

***In parallel*:** Perform IFA labeling on the initial susceptibility phenotype plate

Analysis of the initial susceptibility phenotype:
MIC (Donati, inclusion number) is determined. Interpretation is performed according to the definition of Donati et al. (2010) and Wanninger et al. (2016. PLoS One 11(11): e0166917. doi: 10.1371/journal.pone.0166917) and Suchland et al. (2003)

a. Resistant strains: MIC ≥ 4 µg/ml
b. Sensitive strains: MIC < 2 µg/ml
c. Intermediate strains: 2 µg/ml ≤ MIC < 4 µg/ml

5. Day 5 (sample collection for recovery assay); at 96 hpi
   - Scrape infected cells (marked in yellow below) into the supernatant and freeze at -80°C until use
   - In total, eight samples are taken for each strain (including mock)
     ⇒ low (0.03 or 0.125 µg/ml), intermediate (0.5 µg/ml), high (2 µg/ml) concentrations

Concentrations used in this study:

Mock	8	4	2	1	0.5	0.25	0.125	0.06	0.03	0.015	7.8E-3
Mock	8	4	2	1	0.5	0.25	0.125	0.06	0.03	0.015	7.8E-3

Alternatively, the following samples could be collected:

Mock	8	4	2	1	0.5	0.25	0.125	0.06	0.03	0.015	7.8E-3
Mock	8	4	2	1	0.5	0.25	0.125	0.06	0.03	0.015	7.8E-3

2.3 Recovery assay (Part II)

*Protocol (continuation of Part I)**
Important note concerning medium: Chlamydia cultivation medium* ***contains* *cycloheximide.*

1. *1-3 days prior to start* - Preparation of monolayers:
   Seed 4x 24-well plates for a confluent monolayer on glass coverslips

2. Day 1 (inoculation of samples)
   a) Remove cell growth medium and replace with 1 ml of *Chlamydia* cultivation medium
   b) Inoculation according to outline below
      - Inoculation volumes necessarily differ for resistant and sensitive strains (in order to assure countable inclusion numbers for variable infectivity amongst samples with the plating scheme used herein), therefore initial susceptibility phenotype has to be analyzed before recovery assay (Part II) is performed
      - The IFU/ml of each scraped sample was determined twice (technical duplicats)

Tetracycline resistant strain: Inoculation volume (green = recovery; red = continued exposure)

Mock<br>1 µl	2 µg/ml<br>60 µl	0.5 µg/ml<br>1 µl	0.03 µg/ml<br>1 µl		*Alt.*: 0.125 µg/ml<br>1 µl
Mock<br>1 µl	2 µg/ml<br>60 µl	0.5 µg/ml<br>1 µl	0.03 µg/ml<br>1 µl		0.125 µg/ml<br>1 µl
Mock<br>1 µl	2 µg/ml<br>60 µl	0.5 µg/ml<br>10 µl	0.03 µg/ml<br>1 or 10 µl		0.125 µg/ml<br>10 µl
Mock<br>1 µl	2 µg/ml<br>60 µl	0.5 µg/ml<br>10 µl	0.03 µg/ml<br>1 or 10 µl		0.125 µg/ml<br>10 µl

Tetracycline sensitive strain: Inoculation volume (green = recovery; red = continued exposure)

Mock<br>1 µl	2 µg/ml<br>60 µl	0.5 µg/ml<br>60 µl	0.03 µg/ml<br>1 µl		*Alt.*: 0.125 µg/ml<br>10 µl
Mock<br>1 µl	2 µg/ml<br>60 µl	0.5 µg/ml<br>60 µl	0.03 µg/ml<br>1 µl		0.125 µg/ml<br>10 µl
Mock<br>1 µl	2 µg/ml<br>60 µl	0.5 µg/ml<br>60 µl	0.03 µg/ml<br>60 µl		0.125 µg/ml<br>60 µl
Mock<br>1 µl	2 µg/ml<br>60 µl	0.5 µg/ml<br>60 µl	0.03 µg/ml<br>60 µl		0.125 µg/ml<br>60 µl

   c) Centrifuge plates for 1 h, 1000 g, 25 °C
   d) After centrifugation, wash "continued exposure" two times with *Chlamydia* cultivation medium to remove residual drugs
   e) Replace all inocula with 1 ml of *Chlamydia* cultivation medium
   f) Incubate plates for 34 h at 37 °C (5% CO_2)

3. Day 3 (fixation of plates for recovery assay)
   a) Remove culture medium and fix plates in methanol (cooled to -20 °C, 10 min)
   b) Replace methanol with PBS and store at 4 °C until IFA labeling is carried out

Analysis of the recovery assay:
Calculate the mean IFU/ml for each sample from the technical duplicates. Graph and compare all values (log scale for y-axis) and create a table from parameters as described in "Important definitions and parameters"

a. *Resistance potential* (exp to mock)
b. *Recovery potential* (rec to mock)
c. *Survival after continued exposure* (exp to rec)

**1) Resistance potential**	**Resistant example (SWA-141)**	**Sensitive example (S45/6)**
>25%	0.5 µg/ml	<0.03 µg/ml
>10%	0.5 µg/ml	0.03 µg/ml
**2) Recovery potential**	**SWA-141**	**S45/6**
>10%	0.5 µg/ml	0.03 µg/ml
>1%	2 µg/ml	0.03 µg/ml
**3) Survival after exp**	**SWA-141**	**S45/6**
>10%	2 µg/ml	0.03 µg/ml
>1%	2 µg/ml	0.03 µg/ml
Interpretation	***resistant***	***sensitive***

2.4 MIC confirmation

Up to 4 strains can be tested in one 24-well plate. A tetracycline resistant (e.g. R19 (Dugan et al. 2004), SWA-141) and a tetracycline sensitive control (e.g. S45/6, SWA-14, SWA-86) should be included.

*Protocol**
Important note concerning medium: Chlamydia cultivation medium* ***contains* *cycloheximide.*

1. *1-3 days prior to start* - Preparation of monolayers:
   Seed 1x 24-well plate for a confluent monolayer on glass coverslips

2. Day 1 (start infection)
   a) Prepare a chlamydial suspension with an MOI of 0.1 to infect 4 wells per strain (1 ml)
   b) Aspirate cell growth medium, add **1000 µl** of chlamydial suspension per well:

Mock	Mock	Mock	Mock	Mock	Mock
½ MIC	½ MIC	½ MIC	½ MIC	½ MIC	½ MIC
MIC*	MIC*	MIC*	MIC*	MIC*	MIC*
2x MIC	2x MIC	2x MIC	2x MIC	2x MIC	2x MIC

   *MIC as determined in the initial susceptibility phenotype
   Gray: sensitive control; blue to purple: strains 1-4; black: resistant control
   c) Centrifuge plates for 1 h, 1000 g, 25 °C
   d) Replace all inocula with 1 ml of *Chlamydia* cultivation medium with appropriate tetracycline concentrations, as shown, corresponding to the intitial phenotype
   e) Incubate plates for 34 h at 37 °C (5% CO_2)

3. Day 2 (fixation of plates for MIC confirmation); at 34 hours post infection (hpi)*
   *Alternatively, incubation for 48 h is possible as performed for SULF and PenG in this study
   a) Remove culture medium and fix plates in methanol (cooled to -20 °C, 10 min)
   b) Replace methanol with PBS and store at 4 °C until IFA labeling is carried out

Analysis of MIC confirmation:
MIC (Donati, inclusion number) and MIC (Suchland, inclusion size/morphology) is determined according to "Immunofluorescence analysis" (this protocol, page 2). Interpretation is performed according to the definition of Donati et al. (Donati et al. 2010.; Wanninger et al. 2016.) for MIC (Donati) and according to Suchland et al. (2003) for MIC (Suchland).

a. Resistant strains: MIC ≥ 4 µg/ml
b. Sensitive strains: MIC < 2 µg/ml
c. Intermediate strains: 2 µg/ml ≤ MIC < 4 µg/ml

Create a table to summarize all MIC data to show the consensus MIC and interpretation. Shown is an example (Table 2 of this study):

**Week 1**	**SWA-14**	**SWA-86**	**SWA-141**	**S45/6**
MIC (Donati)	0.03 µg/ml	0.03 µg/ml	4 µg/ml	0.06 µg/ml
**Week 2**	**SWA-14**	**SWA-86**	**SWA-141**	**S45/6**
MIC (Donati)	0.06 µg/ml	0.03 µg/ml	4 µg/ml	0.125 µg/ml
MIC (Suchland)	0.06 µg/ml	0.03 µg/ml	4 µg/ml	0.06-0.125 µg/ml
**MIC consensus**	**0.03-0.06 µg/ml**	**0.03 µg/ml**	**4 µg/ml**	**0.06-0.125 µg/ml**
Interpretation	***sensitive***	***sensitive***	***resistant***	***sensitive***

The entire assay should be repeated for strains where the MIC in Week 1 and 2 is considerably different. If the difference persists upon repetition, an additional analysis should be performed comparing cycloheximide-free immediate seed&infection (initial susceptibility phenotype) with cycloheximide-containing infection of prepared monolayers (MIC confirmation) in a 24- well plate: Tetracycline concentration range 0.03-8 µg/ml (2x 10 wells including mock for both conditions).

3 Sulfamethoxazole (SULF)*

*Considering the initial susceptibility phenotype, this is an example of an antibiotic for which the susceptibility range of *Chlamydia* was previously unknown. For the recovery assay, chosen samples (low, intermediate, high concentration) depend on the specific susceptibility range per drug (which was high for sulfamethoxazole).

3.1 Initial susceptibility phenotype / Recovery assay (Part I)

Up to 4 strains can be tested in three 96-well plates.

*Protocol**
Important note concerning medium: Chlamydia cultivation medium* ***does not contain* *cycloheximide.*

1. Day 1 (seed & infection)
   Preparation of SULF dilutions for the initial susceptibility phenotype and the recovery assay (Part I):
   a) Prepare sulfamethoxazole dilutions ⇒ 2048 µg/ml sulfamethoxazole [102.4 µl stock (50 mg/ml) in 2397.6 µl *Chlamydia* cultivation medium]
   b) Prepare sulfamethoxazole dilution in 96-well plates as shown below
      Dilutions are prepared fresh every time (not stored frozen). Discard 100 µl from 9.8E-04 µg/ml.

Initial susceptibility phenotype / recovery assay (Day 1; n=3): Perform 2-fold dilution with a multichannel pipet

Well	1	2	3	4	5	6	7	8	9	10	11	12
**Conc. (µg/ml)**<br>**SULF**<br>**Medium)**	Mock<br>None<br>100 µl	2048<br>200 µl<br>0 µl	1024<br>100 µl<br>100 µl	512<br>100 µl<br>100 µl	256<br>100 µl<br>100 µl	128<br>100 µl<br>100 µl	64<br>100 µl<br>100 µl	32<br>100 µl<br>100 µl	16<br>100 µl<br>100 µl	8<br>100 µl<br>100 µl	4<br>100 µl<br>100 µl	2<br>100 µl<br>100 µl
**Conc. (µg/ml)**<br>**SULF**<br>**Medium)**	1<br>100 µl<br>100 µl	0.5<br>100 µl<br>100 µl	0.25<br>100 µl<br>100 µl	0.125<br>100 µl<br>100 µl	0.06<br>100 µl<br>100 µl	0.03<br>100 µl<br>100 µl	0.015<br>100 µl<br>100 µl	7.8E-3<br>100 µl<br>100 µl	3.9E-3<br>100 µl<br>100 µl	2.0E-3<br>100 µl<br>100 µl	9.8E-4<br>100 µl<br>100 µl	Mock<br>None<br>100 µl
**Conc. (µg/ml)**<br>**SULF**<br>**Medium)**	Mock<br>None<br>100 µl	2048<br>200 µl<br>0 µl	1024<br>100 µl<br>100 µl	512<br>100 µl<br>100 µl	256<br>100 µl<br>100 µl	128<br>100 µl<br>100 µl	64<br>100 µl<br>100 µl	32<br>100 µl<br>100 µl	16<br>100 µl<br>100 µl	8<br>100 µl<br>100 µl	4<br>100 µl<br>100 µl	2<br>100 µl<br>100 µl
**Conc. (µg/ml)**<br>**SULF**<br>**Medium)**	1<br>100 µl<br>100 µl	0.5<br>100 µl<br>100 µl	0.25<br>100 µl<br>100 µl	0.125<br>100 µl<br>100 µl	0.06<br>100 µl<br>100 µl	0.03<br>100 µl<br>100 µl	0.015<br>100 µl<br>100 µl	7.8E-3<br>100 µl<br>100 µl	3.9E-3<br>100 µl<br>100 µl	2.0E-3<br>100 µl<br>100 µl	9.8E-4<br>100 µl<br>100 µl	Mock<br>None<br>100 µl
**Conc. (µg/ml)**<br>**SULF**<br>**Medium)**	Mock<br>None<br>100 µl	2048<br>200 µl<br>0 µl	1024<br>100 µl<br>100 µl	512<br>100 µl<br>100 µl	256<br>100 µl<br>100 µl	128<br>100 µl<br>100 µl	64<br>100 µl<br>100 µl	32<br>100 µl<br>100 µl	16<br>100 µl<br>100 µl	8<br>100 µl<br>100 µl	4<br>100 µl<br>100 µl	2<br>100 µl<br>100 µl
**Conc. (µg/ml)**<br>**SULF**<br>**Medium)**	1<br>100 µl<br>100 µl	0.5<br>100 µl<br>100 µl	0.25<br>100 µl<br>100 µl	0.125<br>100 µl<br>100 µl	0.06<br>100 µl<br>100 µl	0.03<br>100 µl<br>100 µl	0.015<br>100 µl<br>100 µl	7.8E-3<br>100 µl<br>100 µl	3.9E-3<br>100 µl<br>100 µl	2.0E-3<br>100 µl<br>100 µl	9.8E-4<br>100 µl<br>100 µl	Mock<br>None<br>100 µl
**Conc. (µg/ml)**<br>**SULF**<br>**Medium)**	Mock<br>None<br>100 µl	2048<br>200 µl<br>0 µl	1024<br>100 µl<br>100 µl	512<br>100 µl<br>100 µl	256<br>100 µl<br>100 µl	128<br>100 µl<br>100 µl	64<br>100 µl<br>100 µl	32<br>100 µl<br>100 µl	16<br>100 µl<br>100 µl	8<br>100 µl<br>100 µl	4<br>100 µl<br>100 µl	2<br>100 µl<br>100 µl
**Conc. (µg/ml)**<br>**SULF**<br>**Medium)**	1<br>100 µl<br>100 µl	0.5<br>100 µl<br>100 µl	0.25<br>100 µl<br>100 µl	0.125<br>100 µl<br>100 µl	0.06<br>100 µl<br>100 µl	0.03<br>100 µl<br>100 µl	0.015<br>100 µl<br>100 µl	7.8E-3<br>100 µl<br>100 µl	3.9E-3<br>100 µl<br>100 µl	2.0E-3<br>100 µl<br>100 µl	9.8E-4<br>100 µl<br>100 µl	Mock<br>None<br>100 µl

**NOTE: Shaded wells indicate the minimum number of wells required for evaluation of a single strain.*

   a) Split cells into cycloheximide-free medium and count cells to prepare a cell suspension with **300,000 cells/ml** (30,000 cells per well)
   b) Add chlamydial stock for a final MOI of 0.5
   c) Add 100 µl of Chlamydia/cell mix per well to 100 µl prepared antibiotics (see below)
   d) Centrifugation: 1 h, 1000 g, 25 °C
   e) Incubation: 37 °C, 5% CO_2

Initial susceptibility phenotype: Final sulfamethoxazole concentration (µg/ml)

Mock	1024	512	256	128	64	32	16	8	4	2	1
0.5	0.25	0.13	0.06	0.03	0.015	7.8E-3	3.9E-3	2.0E-3	9.8E-4	4.9E-4	Mock
Mock	1024	512	256	128	64	32	16	8	4	2	1
0.5	0.25	0.13	0.06	0.03	0.015	7.8E-3	3.9E-3	2.0E-3	9.8E-4	4.9E-4	Mock
Mock	1024	512	256	128	64	32	16	8	4	2	1
0.5	0.25	0.13	0.06	0.03	0.015	7.8E-3	3.9E-3	2.0E-3	9.8E-4	4.9E-4	Mock
Mock	1024	512	256	128	64	32	16	8	4	2	1
0.5	0.25	0.13	0.06	0.03	0.015	7.8E-3	3.9E-3	2.0E-3	9.8E-4	4.9E-4	Mock

Blue to purple: strains 1-4

Recovery Assay 1: Final sulfamethoxazole concentration (µg/ml)

Mock	1024	512	256	128	64	32	16	8	4	2	1
0.5	0.25	0.13	0.06	0.03	0.015	7.8E-3	3.9E-3	2.0E-3	9.8E-4	4.9E-4	Mock
Mock	1024	512	256	128	64	32	16	8	4	2	1
0.5	0.25	0.13	0.06	0.03	0.015	7.8E-3	3.9E-3	2.0E-3	9.8E-4	4.9E-4	Mock
Mock	1024	512	256	128	64	32	16	8	4	2	1
0.5	0.25	0.13	0.06	0.03	0.015	7.8E-3	3.9E-3	2.0E-3	9.8E-4	4.9E-4	Mock
Mock	1024	512	256	128	64	32	16	8	4	2	1
0.5	0.25	0.13	0.06	0.03	0.015	7.8E-3	3.9E-3	2.0E-3	9.8E-4	4.9E-4	Mock

Blue & red: strains 1&2 in duplicate (for rec [top rows], exp [bottom rows])

Recovery Assay 2: Final sulfamethoxazole concentration (µg/ml)

Mock	1024	512	256	128	64	32	16	8	4	2	1
0.5	0.25	0.13	0.06	0.03	0.015	7.8E-3	3.9E-3	2.0E-3	9.8E-4	4.9E-4	Mock
Mock	1024	512	256	128	64	32	16	8	4	2	1
0.5	0.25	0.13	0.06	0.03	0.015	7.8E-3	3.9E-3	2.0E-3	9.8E-4	4.9E-4	Mock
Mock	1024	512	256	128	64	32	16	8	4	2	1
0.5	0.25	0.13	0.06	0.03	0.015	7.8E-3	3.9E-3	2.0E-3	9.8E-4	4.9E-4	Mock
Mock	1024	512	256	128	64	32	16	8	4	2	1
0.5	0.25	0.13	0.06	0.03	0.015	7.8E-3	3.9E-3	2.0E-3	9.8E-4	4.9E-4	Mock

Blue & red: strains 1&2 in duplicate (for rec [top], exp [bottom])

2. Day 2 (fixation of plates for initial susceptibility phenotype); at 34 hours post infection (hpi)
   a) Remove culture medium and fix plates in methanol (cooled to -20 °C, 10 min)
   b) Replace methanol with PBS and store at 4 °C until IFA labeling is carried out

3. Day 3 (wash plates for recovery assay & analysis of initial susceptibility phenotype); at 48 hpi
   a) Prepare SULF dilutions (white = recovery; blue = continued exposure):

None	None	None	None	None	None	None	None	None	None	None	None
None	None	None	None	None	None	None	None	None	None	None	None
Mock	1024	512	256	128	64	32	16	8	4	2	1
0.5	0.25	0.13	0.06	0.03	0.015	7.8E-3	3.9E-3	2.0E-3	9.8E-4	4.9E-4	Mock

   b) Wash the recovery plate ("Day 1") 2x with *Chlamydia* cultivation medium
   c) Transfer the media from the "Recovery assay – Day 3 plate" to recovery plates
   d) Incubation of plate at 37 °C (5% CO_2)

***In parallel*:** Perform the immunofluorescence assay on the initial susceptibility phenotype plate according to the protocol described above ("IFA labeling")

Analysis of the initial susceptibility phenotype:

MIC (Donati, inclusion number) is determined. If that is not possible because there is no reduction regarding the inclusion number (e.g. for sulfamethoxazole), MIC (Suchland, inclusion size/morphology) is determined.

4. Friday (Day 5; sample collection for recovery assay); at 96 hpi
   - Scrape wells (marked in yellow below) into medium and freeze at -80°C until use
   - In total, eight samples are taken for each strain (including mock) – low, intermediate, high

Specific for sulfamethoxazole in this study (large susceptibility range; no inclusion number reduction)

Mock	1024	512	256	128	64	32	16	8	4	2	1
0.5	0.25	0.13	0.06	0.03	0.015	7.8E-3	3.9E-3	2.0E-3	9.8E-4	4.9E-4	Mock

For this study, additional samples were taken and analyzed (Supplementary Figure 3B)

3.2 Recovery assay (Part II)

*Protocol (continuation of Part I)**
Important note concerning medium: Chlamydia cultivation medium **contains cycloheximide.*

1. *1-3 days prior to start* - Preparation of monolayers:
   Seed 4x 24-well plates for a confluent monolayer on glass coverslips

2. Day 1 (inoculation of samples)
   g) Remove cell growth medium and replace with 1 ml of *Chlamydia* cultivation medium
   h) Inoculation according to outline below
      - Inoculation volumes differ for resistant and sensitive strains, therefore initial susceptibility phenotype has to be analyzed before recovery assay (Part II) is performed
      - Perform technical duplicates of each sample

Resistant strains (MIC > 64 µg/ml; e.g. SWA-14, 86, 141), rec = green; exp = red

Mock<br>1 µl	512 µg/ml<br>60 µl	128 µg/ml<br>60 µl	2 µg/ml<br>1 µl	*Add.* 32 µg/ml<br>10 µl	*Add.* 8 µg/ml<br>1 µl
Mock<br>1 µl	512 µg/ml<br>60 µl	128 µg/ml<br>60 µl	2 µg/ml<br>1 µl	*Add.* 32 µg/ml<br>10 µl	*Add.* 8 µg/ml<br>1 µl
Mock<br>1 µl	512 µg/ml<br>60 µl	128 µg/ml<br>60 µl	2 µg/ml<br>1 µl	*Add.* 32 µg/ml<br>60 µl	*Add.* 8 µg/ml<br>10 µl
Mock<br>1 µl	512 µg/ml<br>60 µl	128 µg/ml<br>60 µl	2 µg/ml<br>1 µl	*Add.* 32 µg/ml<br>60 µl	*Add.* 8 µg/ml<br>10 µl

Sensitive strains (MIC < 1 µg/ml; e.g. S45/6)*, rec = green; exp = red

Mock<br>1 µl	512 µg/ml<br>60 µl	128 µg/ml<br>60 µl	2 µg/ml<br>60 µl	*Add.* 32 µg/ml<br>60 µl	*Add.* 8 µg/ml<br>60 µl
Mock<br>1 µl	512 µg/ml<br>60 µl	128 µg/ml<br>60 µl	2 µg/ml<br>60 µl	*Add.* 32 µg/ml<br>60 µl	*Add.* 8 µg/ml<br>60 µl
Mock<br>1 µl	512 µg/ml<br>60 µl	128 µg/ml<br>60 µl	2 µg/ml<br>60 µl	*Add.* 32 µg/ml<br>60 µl	*Add.* 8 µg/ml<br>60 µl
Mock<br>1 µl	512 µg/ml<br>60 µl	128 µg/ml<br>60 µl	2 µg/ml<br>60 µl	*Add.* 32 µg/ml<br>60 µl	*Add.* 8 µg/ml<br>60 µl

*MIC S45/6 = 0.0039 to 0.0078 µg/ml; 60 µl inoculation for volume for all samples at ≥MIC

   i) Centrifugation 1 h, 1000g, 25 °C
   j) Wash "exp" two times with chlaymdiae cultivation medium to remove residual drugs
   k) Replace all inocula with 1 ml of *Chlamydia* cultivation medium
   l) Incubation of plate at 37 °C (5% CO_2)

4. Day 3 (fixation of plates for recovery assay)
   a) Remove culture medium and fix plates in methanol (cooled to -20 °C, 10 min)
   b) Replace methanol with PBS and store at 4 °C until IFA labeling is carried out

Analysis of the recovery assay:
Calculate the mean IFU/ml for each sample from the technical duplicates. Create a bar graph comparing all values (log scale for y-axis) and create a table from parameters as described in "Important definitions and parameters"

a. *Resistance potential* (exp to mock)
b. R*ecovery potential* (rec to mock)
c. *Survival after continued exposure* (exp to rec)

**a) Resistance potential**	**Resistant example**	**Sensitive example**
>25%	2 µg/ml	< 3.9E-3 µg/ml
>10%	2 µg/ml	< 3.9E-3 µg/ml
**b) Recovery potential**	**SWA-141**	**S45/6**
>10%	2 µg/ml	< 3.9E-3 µg/ml
>1%	32 µg/ml	3.9E-3 µg/ml
**c) Survival after exp**	**SWA-141**	**S45/6**
>10%	2 µg/ml	< 3.9E-3 µg/ml
>1%	2 µg/ml	3.9E-3 µg/ml
Interpretation	***resistant***	***sensitive***

3.3 MIC confirmation

Up to 4 strains can be tested in one 24-well plate.

*Protocol**

Important note concerning medium: Chlamydia cultivation medium* ***contains* *cycloheximide.*

1. *1-3 days prior to start* - Preparation of monolayers:
   Seed 1x 24-well plate for a confluent monolayer on glass coverslips

2. Day 1 (start infection)
   c) Prepare a chlamydial suspension with an MOI of 0.1 to infect 4 wells per strain (1 ml)
   d) Aspirate cell growth medium, add **1000 µl** of chlamydial suspension per well:

Mock	Mock	Mock	Mock	(**)	
½ MIC	½ MIC	½ MIC	½ MIC		
MIC*	MIC*	MIC*	MIC*		
2x MIC	2x MIC	2x MIC	2x MIC		

   *MIC as determined in the initial susceptibility phenotype
   (**) Additional sulfamethoxazole concentrations available
   In this study, in addition to 0.0039, 0.0078 and 0.015 µg/ml (MIC ± 2-fold dilution), the following concentrations were analyzed for S45: 0.06, 0.25, 1, 4, 16, 64, 128, 256 µg/ml (64-256 µg/ml was used for the field strains SWA-14, 86, 141)
   e) Centrifugation: 1 h, 1000g, 25 °C
   f) *During centrifugation*: Prepare antibiotic dilution
   g) Add 1 ml of cultivation medium with/without appropriate SULF concentration
   h) Incubation: 37 °C, 5% CO_2

3. Day 2 (fixation of plates for MIC confirmation); at 34 hours post infection (hpi)*
   *Alternatively, incubation for 48 hours is possible as performed in this study
   a) Remove culture medium and fix plates in methanol (cooled to -20 °C, 10 min)
   b) Replace methanol with PBS and store at 4 °C until IFA labeling is carried out

Analysis of MIC confirmation:

MIC (Donati, inclusion number) analysis was not possible for sulfamethoxazole because the inclusion number was not altered. MIC (Suchland, inclusion size/morphology) is determined according to "Immunofluorescence analysis" (this protocol, page 2).

There is no published definition for resistance or susceptibility to sulfamethoxazole. In this study, there was a clear difference between the field strains and S45/6, which we interpreted and reported as resistant and sensitive, respectively.

Create a table to summarize all MIC data to show the consensus MIC and interpretation. Shown is an example (Table 4 of this study):

**Sulfamethoxazole**	**SWA-14**	**SWA-86**	**SWA-141**	**S45/6**
Initial susceptibility phenotype	128-256 µg/ml	64-128 µg/ml	128-256 µg/ml	3.9E-3 µg/ml
MIC confirmation	256 µg/ml	64-128 µg/ml	128-256 µg/ml	7.8E-3 µg/ml
**MIC consensus**	**128-256 µg/ml**	**64-128 µg/ml**	**128-256 µg/ml**	**3.9-7.8E-3 µg/ml**
Interpretation	***resistant***	***resistant***	***resistant***	***Sensitive***

4 Penicillin G (PenG)

4.1 <u>Initial susceptibility phenotype / Recovery assay (Part I)</u>

Up to 4 strains can be tested in three 96-well plates.

*Protocol**
Important note concerning medium: Chlamydia cultivation medium* ***does not contain* *cycloheximide.*

1. Day 1 (seed & infection); perform in the morning
   PenG dilutions & seed/infection for the initial susceptibility phenotype and the recovery assay (Part I):
   a) Prepare a solution with 200 U/ml and perform a 1 :10 dilution (0.002-200 U/ml)
   b) Add 100 µl of antibiotic to 96-well plate (row 1 = mock; row 2-6 = descending PenG)
   c) Split cells into cycloheximide-free medium and count cells to prepare a cell suspension with **300,000 cells/ml** (30,000 cells per well), 2 plates
   d) Add chlamydial stock for a final MOI of 0.5
   e) Add 100 µl of Chlamydia/cell mix per well to 100 µl prepared antibiotics (see below)
   f) Centrifugation: 1 h, 1000 g, 25 °C
   g) Incubation: 37 °C, 5% CO_2

   Final PenG concentration post seed&infection for each strain:

**1**	**2**	**3**	**4**	**5**	**6**	**7**
<u>Mock</u>	<u>100 U/ml</u>	<u>10 U/ml</u>	<u>1 U/ml</u>	<u>0.1 U/ml</u>	<u>0.01 U/ml</u>	<u>0.001 U/ml</u>

2. Day 2 (fixation of plate for initial susceptibility phenotype); at 34 hours post infection (hpi)
   a) Remove culture medium and fix plates in methanol (cooled to -20 °C, 10 min)
   b) Replace methanol with PBS and store at 4 °C until IFA labeling is carried out

3. Day 3 (wash plates for recovery assay & analysis of initial susceptibility phenotype); at 48 hpi
   - Prepare PenG dilutions (white = recovery; blue = continued exposure):

**None**	**None**	**None**	**None**	**None**	**None**	**None**
**Mock**	**100**	**10**	**1**	**0.1**	**0.01**	**0.001**

   - Wash the recovery plate ("Day 1") 2x with *Chlamydia* cultivation medium
   - Transfer the media from the "Recovery assay – Day 3 plate" to the recovery plate
   - Incubation of plate at 37 °C (5% CO_2)

<u>In parallel</u>**:** Perform the immunofluorescence assay on the initial susceptibility phenotype plate according to the protocol described above ("IFA labeling")

<u>Analysis of the initial susceptibility phenotype:</u>
Check at what concentration the first aberrant inclusions are visible. Determine the approximate proportion of aberrant to normal inclusions.

5. Day 5 (sample collection for recovery assay); at 96 hpi
   - Scrape wells (marked in yellow below) into medium and freeze at -80°C until use
   - In total, eight samples are taken for each strain (including mock)

**Mock**	**100**	**10**	**1**	**0.1**	**0.01**	**0.001**

4.2 Recovery assay (Part II)

*Protocol (continuation of Part I)**
Important note concerning medium: Chlamydia cultivation medium **contains cycloheximide.*

1. *1-3 days prior to start* - Preparation of monolayers:
   Seed 4x 24-well plates for a confluent monolayer on glass coverslips

2. Day 1 (inoculation of samples)
   a) Remove cell growth medium and replace with 1 ml of *Chlamydia* cultivation medium
   b) Inoculation according to outline below
      - Inoculation volumes differ for resistant and sensitive strains, therefore initial susceptibility phenotype has to be analyzed before recovery assay (Part II) is performed
      - Perform technical duplicates of each sample

Mock<br>1 µl	100 U/ml<br>60 µl	100 U/ml<br>60 µl	100 U/ml<br>60 µl
Mock<br>1 µl	100 U/ml<br>60 µl	100 U/ml<br>60 µl	100 U/ml<br>60 µl
Mock<br>1 µl	100 U/ml<br>60 µl	100 U/ml<br>60 µl	100 U/ml<br>60 µl
Mock<br>1 µl	100 U/ml<br>60 µl	100 U/ml<br>60 µl	100 U/ml<br>60 µl

   c) Centrifugation 1 h, 1000g, 25 °C
   d) Wash “exp” two times with chlaymdiae cultivation medium to remove residual drugs
   e) Replace all inocula with 1 ml of *Chlamydia* cultivation medium
   f) Incubation of plate at 37 °C (5% CO_2)

3. Day 3 (fixation of plates for recovery assay)
   a) Remove culture medium and fix plates in methanol (cooled to -20 °C, 10 min)
   b) Replace methanol with PBS and store at 4 °C until IFA labeling is carried out

Analysis of the recovery assay:
Calculate the mean IFU/ml for each sample from the technical duplicates. Create a bar graph comparing all values (log scale for y-axis) and create a table from parameters as described in “Important definitions and parameters”

4.3 MIC confirmation / Inclusion size analysis

Up to 4 strains can be tested in one 24-well plate.

*Protocol**
Important note concerning medium: Chlamydia cultivation medium **contains cycloheximide.*

1. *1-3 days prior to start* - Preparation of monolayers:
   Seed 1x 24-well plate for a confluent monolayer on glass coverslips

2. Day 1 (start infection)
   a) Prepare a chlamydial suspension with an MOI of 0.1 to infect 4 wells per strain (1 ml)
   b) Aspirate cell growth medium, add **1000 µl** of chlamydial suspension per well:

Mock	Mock	Mock	Mock	(**)	
1 U/ml	1 U/ml	1 U/ml	1 U/ml		
10 U/ml	10 U/ml	10 U/ml	10 U/ml		
100 U/ml	100 U/ml	100 U/ml	100 U/ml		

(**) Additional values could be analyzed in these wells

c) Centrifugation: 1 h, 1000g, 25 °C
d) *During centrifugation*: Prepare antibiotic dilution
e) Add 1 ml of cultivation medium with/without appropriate PenG concentration
f) Incubation: 37 °C, 5% CO_2

3. Day 2 (fixation of plates for MIC confirmation); at 34 hours post infection (hpi)*
   *Alternatively, incubation for 48 hours is possible as performed in this study
   a) Remove culture medium and fix plates in methanol (cooled to -20 °C, 10 min)
   b) Replace methanol with PBS and store at 4 °C until IFA labeling is carried out

<u>*Analysis of MIC confirmation/inclusion size analysis:*</u>
Inclusion size is determined as described in “Immunofluorescence analysis” (page 2). Additionally, inclusion morphology is analyzed both at 400x and 1000x magnification under oil immersion in order to document persistent inclusions.
