# Supplementary figures and images for: Evaluating the Antibiotic Susceptibility of Chlamydia – New Approaches for in Vitro Assays
Source: Front Microbiol. 2018 Jul 3;9:1414. doi: 10.3389/fmicb.2018.01414 (PMC6037721; doi:10.3389/fmicb.2018.01414)

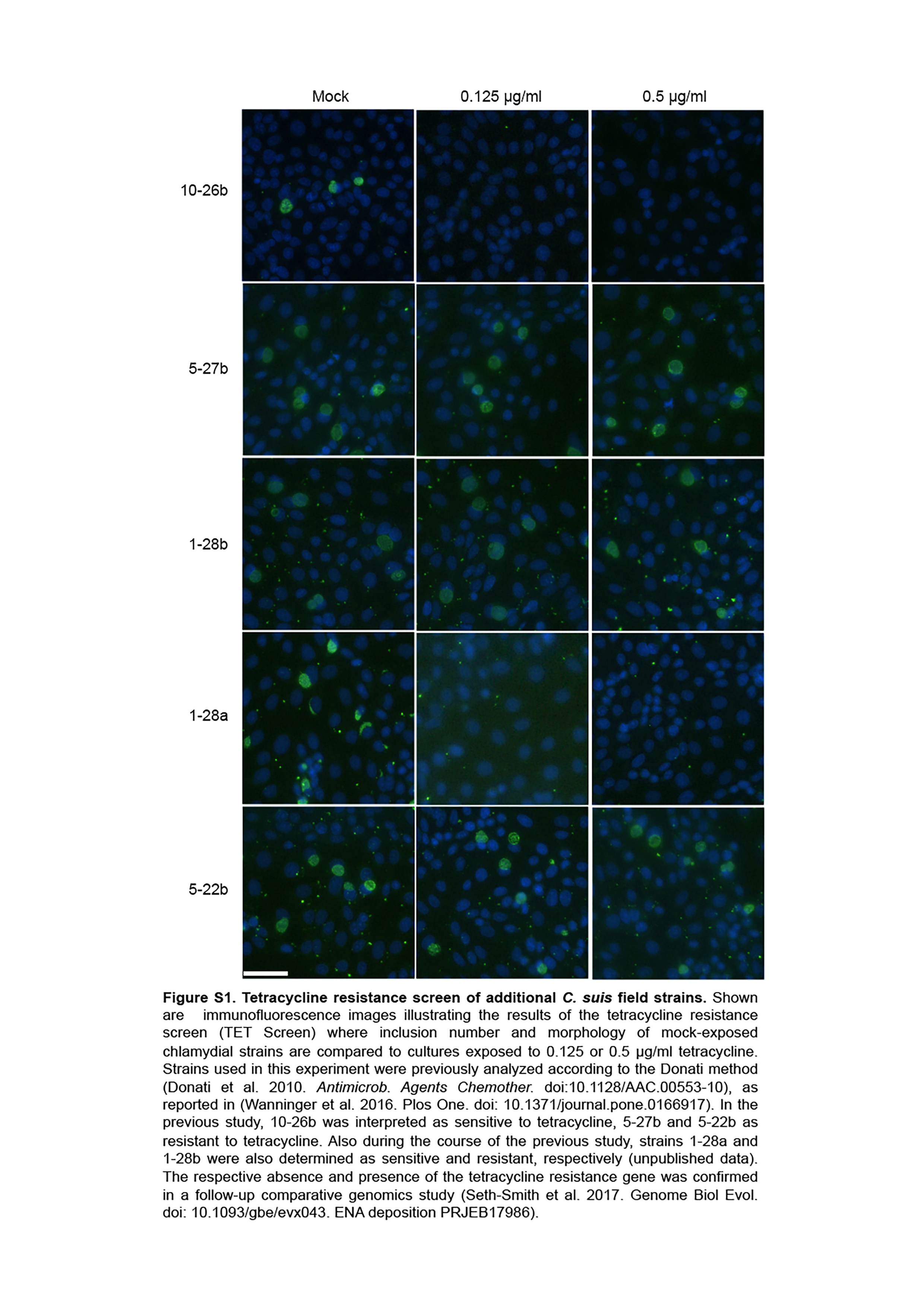

Supplement: Supplementary file 2 [file Image_1.TIF]

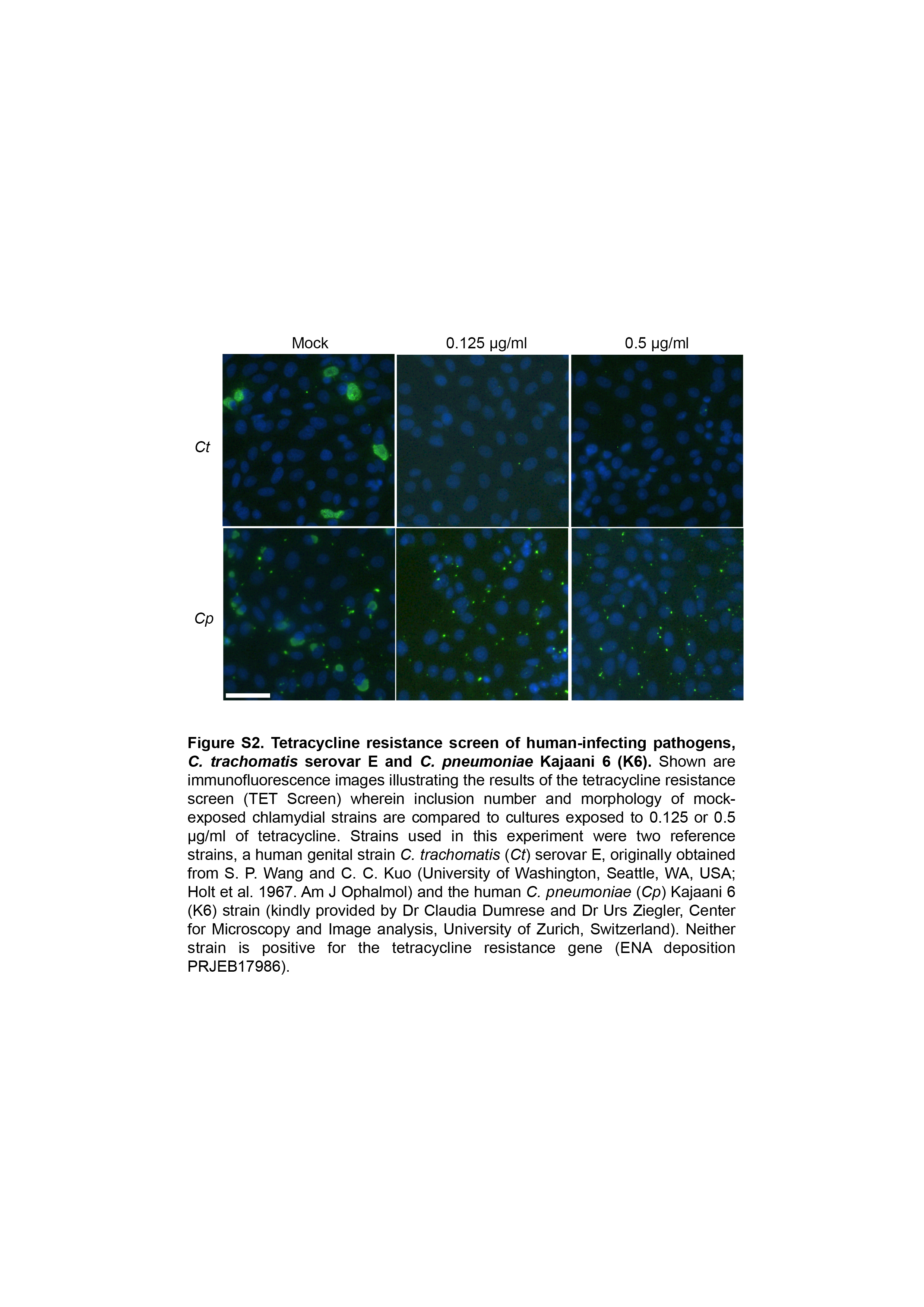

Supplement: Supplementary file 3 [file Image_2.TIF]

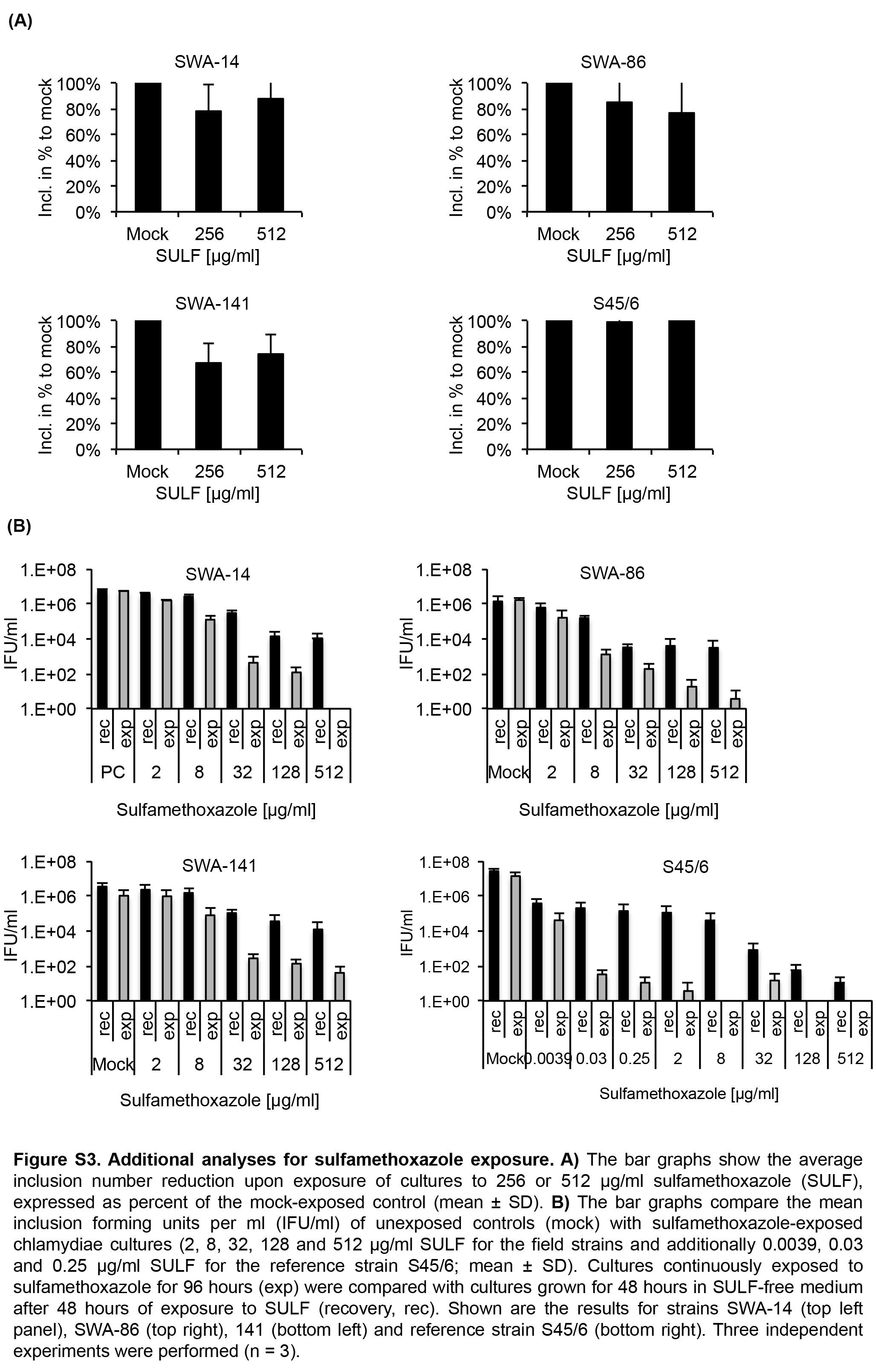

Supplement: Supplementary file 4 [file Image_3.TIF]

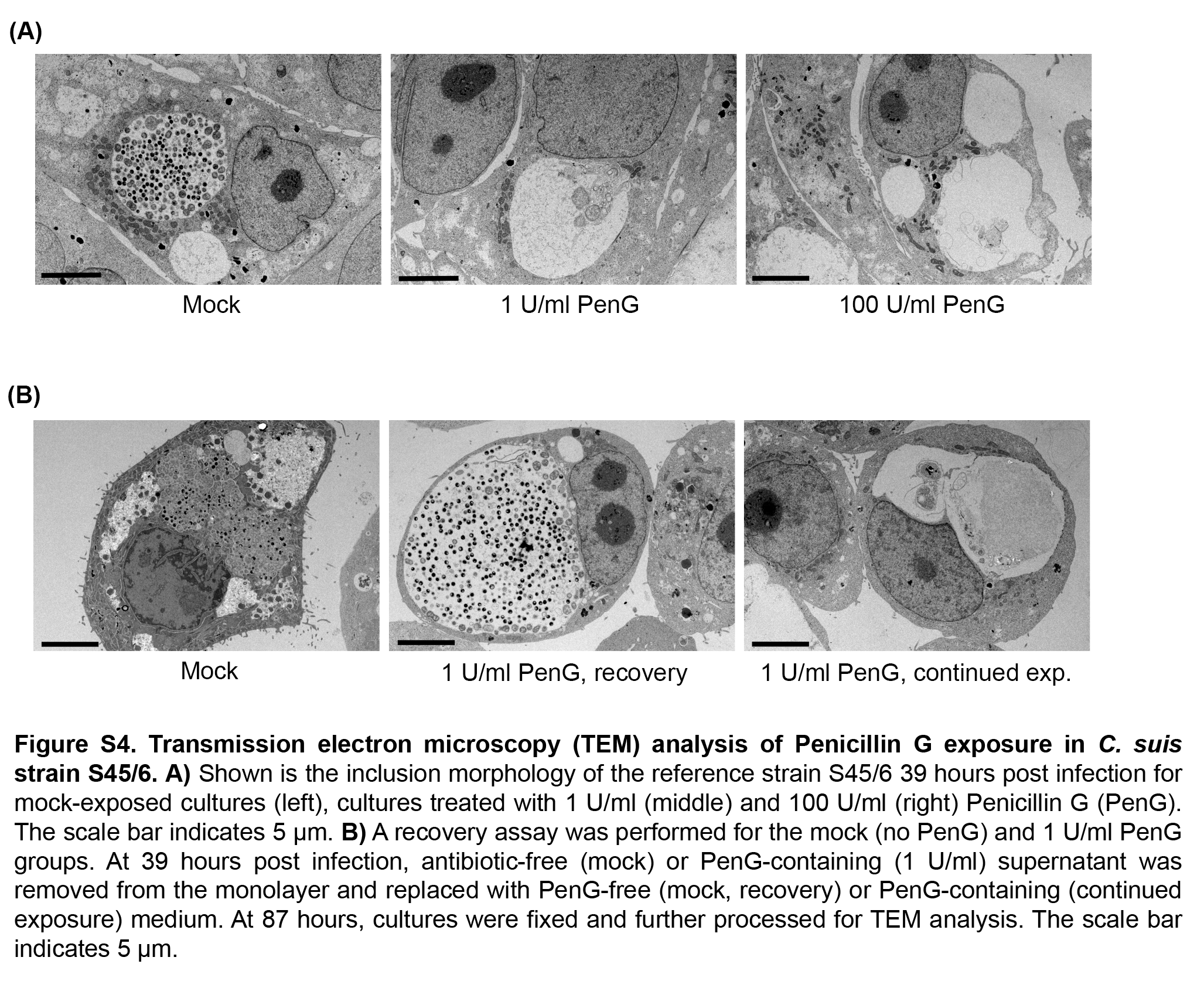

Supplement: Supplementary file 5 [file Image_4.TIF]
